# Formation of the Long Range Dpp Morphogen Gradient

**DOI:** 10.1371/journal.pbio.1001111

**Published:** 2011-07-26

**Authors:** Gerald Schwank, Sascha Dalessi, Schu-Fee Yang, Ryohei Yagi, Aitana Morton de Lachapelle, Markus Affolter, Sven Bergmann, Konrad Basler

**Affiliations:** 1 Institute of Molecular Life Sciences, University of Zurich, Zurich, Switzerland; 2 Department of Medical Genetics, University of Lausanne, Lausanne, Switzerland; 3 Swiss Institute of Bioinformatics, Switzerland; 4 Biozentrum der Universität Basel, Basel, Switzerland; Stanford University, United States of America

## Abstract

The TGF-β homolog Decapentaplegic (Dpp) acts as a secreted morphogen in the Drosophila wing disc, and spreads through the target tissue in order to form a long range concentration gradient. Despite extensive studies, the mechanism by which the Dpp gradient is formed remains controversial. Two opposing mechanisms have been proposed: receptor-mediated transcytosis (RMT) and restricted extracellular diffusion (RED). In these scenarios the receptor for Dpp plays different roles. In the RMT model it is essential for endocytosis, re-secretion, and thus transport of Dpp, whereas in the RED model it merely modulates Dpp distribution by binding it at the cell surface for internalization and subsequent degradation. Here we analyzed the effect of receptor mutant clones on the Dpp profile in quantitative mathematical models representing transport by either RMT or RED. We then, using novel genetic tools, experimentally monitored the actual Dpp gradient in wing discs containing receptor gain-of-function and loss-of-function clones. Gain-of-function clones reveal that Dpp binds in vivo strongly to the type I receptor Thick veins, but not to the type II receptor Punt. Importantly, results with the loss-of-function clones then refute the RMT model for Dpp gradient formation, while supporting the RED model in which the majority of Dpp is not bound to Thick veins. Together our results show that receptor-mediated transcytosis cannot account for Dpp gradient formation, and support restricted extracellular diffusion as the main mechanism for Dpp dispersal. The properties of this mechanism, in which only a minority of Dpp is receptor-bound, may facilitate long-range distribution.

## Author Summary

Morphogens are signaling molecules that trigger specific responses in cells in a concentration-dependent manner. The formation of morphogen gradients is essential for the patterning of tissues and organs. Decapentaplegic (Dpp) is the Drosophila homolog of the bone morphogenic proteins in vertebrates and forms a morphogen gradient along the anterior-posterior axis of the Drosophila wing imaginal disc, a single-cell layered epithelium. Dpp determines the growth and final size of the wing disc and serves as an ideal model system to study gradient formation. Despite extensive studies the mechanism by which morphogen gradients are established remains controversial. In the case of Dpp two mechanisms have been postulated, namely extracellular diffusion and receptor-mediated transcytosis. In the first model Dpp is suggested to move by diffusion through the extracellular matrix of a tissue, whereas in the latter model Dpp is transported through the cells by receptor-mediated uptake and re-secretion. In this work we combined novel genetic tools with mathematical modeling to discriminate between the two models. Our results suggest that the Dpp gradient forms following the extracellular diffusion mechanism. Moreover, our data suggest that the majority of the extracellular Dpp is free and not bound to its receptor, a property likely to play a role for the long-range gradient formation.

## Introduction

How embryonic cells acquire positional information is a key question in developmental biology. The concept of morphogen gradients, proposed more than a century ago [1],[2], has received substantial experimental validation over the past decade (reviewed in [3],[4]). Particularly compelling evidence for their existence comes from the identification of secreted proteins that control cell fates in a concentration-dependent manner. Localized production of Wnt, Hedgehog, and TGF-β family members have been described in numerous tissues and organisms. However, despite extensive studies on these molecules, the mechanism of transport through tissues and the properties which determine the range of morphogen movement remain poorly understood and controversial. Here we use the TGF-β family member Decapentaplegic (Dpp) in the Drosophila wing imaginal disc as a model to address these issues.

Dpp is expressed in a stripe of anterior compartment (A) cells along the anteroposterior (A-P) boundary of the wing disc, and forms a concentration gradient along the A-P axis of the wing primordium [5]–[9]. Upon binding to the type I-type II/Thick veins (Tkv)-Punt receptor complex, the intracellular signal transducer Mothers-against-Dpp (Mad) becomes phosphorylated, forms a complex with Medea, and enters the nucleus to inhibit the expression of the transcriptional repressor Brinker (Brk) [10]–[18]. These events convert the Dpp morphogen gradient into an inverse gradient of Brk activity that mediates many of the patterning and growth functions of Dpp ([19]–[21]; reviewed in [22]).

Although the transduction of the Dpp signal and its role in patterning is well understood, the question of how Dpp is dispersed through its target tissue is still unexplained and thus served as a fertile ground for experimentation and speculations (reviewed in [23]–[25]). Several mechanisms for Dpp movement through the wing disc tissue have been proposed. The simplest model assumes that Dpp disperses by passive extracellular diffusion. However, because the effective diffusion coefficient of Dpp in the wing disc is three orders of magnitude lower than that of a similarly sized molecule in water [26], and because a secreted form of GFP fails to form a gradient in wing discs [5], Dpp gradient formation cannot be explained by free diffusion. Thus a “restricted extracellular diffusion” (RED) model, in which Dpp interacts with its receptor and extracellular matrix (ECM) proteins, has been proposed. This model is supported by theoretical [27] and experimental studies [28],[29], which implicate glypicans in the ECM as essential components for Dpp movement.

A completely different mechanism by which Dpp may achieve its long-range distribution is receptor-mediated transcytosis (RMT) [5],[30]. In this model, Dpp does not move through the *extracellular* space, but rather through the *cell bodies* by repeated cycles of endocytosis and re-secretion. First evidence for this model was gathered from analyzing the Dpp gradient in discs containing *shibire* mutant cell clones, in which dynamin-dependent endocytosis is blocked. Entchev et al. (2000) [5] found reduced Dpp levels “behind” such clones (i.e., on the distal side relative to the source), suggesting that Dpp is unable to traverse the mutant cells. Moreover, small lateral clones mutant for *tkv* also appeared to block Dpp movement [5], indicating that transcytosis is receptor-mediated. Although this work has at first been challenged by mathematical modeling and experimental studies [27],[28], the transcytosis mechanism was further backed up by theoretical considerations [31], and by recent work involving FRAP experiments showing that a GFP:Dpp fusion protein is unable to move into a photobleached region when dynamin-dependent endocytosis is blocked [26].

The two models to explain Dpp movement through an epithelium remain unreconciled, and further analysis is required to determine the contribution of extracellular restricted diffusion or receptor-mediated transcytosis to the formation of the Dpp gradient. The controversy over Dpp dispersal is augmented by yet another scenario, in which Dpp moves along actin-based filopodia, termed cytonemes, which directly project from the receiving cells to the producing cells [32],[33]. Experimental evidence for this mechanism, however, remains elusive, as it is not known yet whether the Dpp ligand is associated with these structures or how a gradient would form along these structures.

Biochemical studies suggest that Dpp binds to the type I receptor Tkv with high affinity [17],[34]. Interestingly, all three above mentioned models for Dpp movement rely on the receptor, yet do so in distinct ways. In the restricted diffusion model, interactions between Dpp and its receptor on the cell surface contribute to the immobilization, subsequent uptake, and degradation of the ligand, thereby impeding Dpp dispersal; in the receptor-mediated transcytosis model the receptor plays an essential role in the uptake (endocytosis) and re-secretion (exocytosis) of Dpp, and thereby facilitates Dpp movement; and finally in the basic cytoneme model the receptor is used to ferry Dpp along cytonemes.

Here we set out to exploit the pivotal role that the Dpp receptor plays in these mechanisms and manipulated the receptor levels in cell clones to discriminate between the different models of morphogen gradient formation. We first confirmed in overexpression clones in vivo that Dpp binds to the type I receptor Tkv, but not to the type II receptor Punt. We then analyzed the effect of *tkv* mutant clones on the Dpp gradient, and also compared the experimental data to the computed predictions for the RMT and RED models. While our results challenge the RMT model and are also incompatible with the basic cytoneme mechanism, they are consistent with a RED scenario, in which the majority of Dpp is not bound to Tkv. Hence we suggest that the major mechanism of Dpp distribution is restricted extracellular diffusion.

## Results

### Modeling Dpp Movement through Receptor Mutant Clones

**Figure 1:**
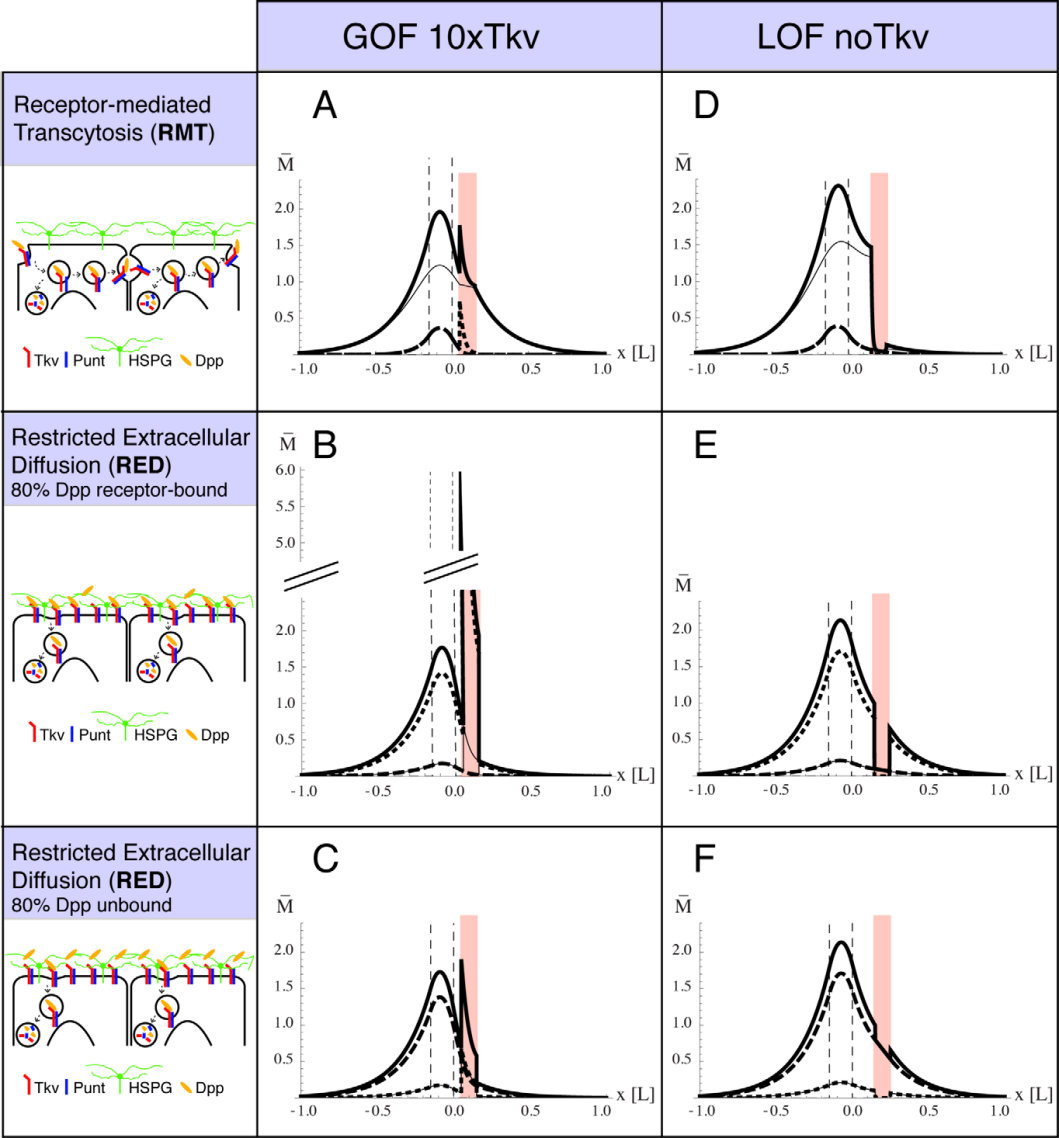
Modeling of the effect of receptor mutant clones on Dpp gradient formation in different transport scenarios. Modeling the effects of GOF (A,B,C) and LOF (D,E,F) receptor mutant clones on Dpp gradient formation in different transport scenarios: RMT (A,D), RED with 80% of Dpp receptor-bound (B,E) and RED with 80% of Dpp unbound (C,F). The position x ranges from −L to L, where L is the half length of the wing pouch. The thick solid line represents the total Dpp profile, the thin solid line internal Dpp, the dotted line receptor-bound Dpp, and the dashed line external unbound Dpp. Dpp levels are always expressed in arbitrary units. The dashed vertical lines show the source boundaries (−15%L and 0), and the clones are located between 5%L to 15%L (A–C) and 15%L to 25%L (D–F). In the GOF clones we assume a 10-fold increase of receptor levels, and in LOF clones we assume nil receptors. GOF clones lead to an increase of Dpp levels inside the clones in all different transport scenarios (A,B,C), and therefore can be distinguished only quantitatively. In LOF clones, however, RMT can also be distinguished qualitatively from the extracellular movement models.

The Dpp receptor plays distinct roles for Dpp dispersal in the “restricted extracellular diffusion” (RED) and “receptor mediated transcytosis” (RMT) models (see Introduction). Thus, the analysis of Dpp gradient formation in a tissue containing receptor mutant clones promises to discriminate between the two models. Here we first chose a theoretical approach to investigate the influence of Dpp receptor mutant clones on the Dpp gradient and quantitatively modeled distinct scenarios representing morphogen transport by either the RMT or the RED model (for a short description of the mathematical modeling, see Box 1; all the analytical details of the model are reported in the Text S1). Three pools of Dpp (external-unbound, receptor-bound and internalized) were described using coupled reaction diffusion equations. This model has a number of free parameters, which could be constrained though by fixing the relative concentrations of the three Dpp pools, and by the approximation that the Dpp profile exponentially decays outside the production region with a decay length of 20 µm [26]. We therefore studied limit case scenarios, in which the relative concentrations of the Dpp pools were fixed and Dpp was either mainly internalized (80% of total Dpp), mainly receptor-bound, or mainly external-unbound (cf. Box 1). Our model involves both, pure external diffusion and receptor-mediated transcytosis [35]. The latter, within its biologically meaningful parameter range (cf. Box 1), only had an important influence on the total Dpp gradient in the limit case scenario in which Dpp was mainly internalized, and it could be neglected in the other two limit case scenarios (cf. Box 1 and Text S1). The RMT model could therefore be represented by the limit case scenario in which Dpp was mainly internalized (Figure 1A and D), and the RED model by the limit case scenarios in which Dpp was mainly receptor-bound (Figure 1B and E) or mainly external (Figure 1C and F).

**Box 1: Modeling the Effect of Receptor Mutant Clones on the Dpp Gradient**

**Modeling Dpp**

We assume that Dpp diffuses in the extracellular medium and binds to the Tkv receptors at the cell surface. Tkv-bound Dpp can then unbind or be internalized. After internalization, Dpp is either degraded or transported to a neighboring cell by transcytosis [5]. We therefore identify three distinct components (pools) contributing to the total Dpp concentration profile: external *M_e_*(*x*), Tkv-bound *M_b_*(*x*), and internalized *M_i_*(*x*), leading to *M_tot_*(*x*) = *M_e_*(*x*)+*M_b_*(*x*)+*M_i_*(*x*). The steady state profile of each component is described by a non-linear ordinary differential equation, yielding
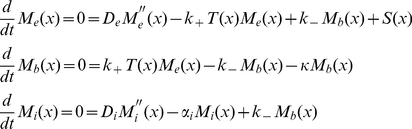
(1)where *D_e_* is the extracellular diffusion constant, *k*_+_ and *k*_−_ are the binding/unbinding rates, *T*(*x*) = *T*_0_−*M* *_b_*(*x*) is the local number of free receptors (with *T*_0_ the homogeneous local total number of receptors), *S*(*x*) describes the source (Dpp production region), *κ* is the internalization rate and *α*_i_ refers to the internal linear degradation. We also assume that transcytosis can be described in a diffusive way by introducing an “effective internal diffusion constant” *D_i_* [35]. Assuming a large number of receptors, i.e. 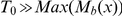, the number of free receptors is almost constant: 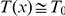. In this case, the set of equations (1) becomes linear and reduces to
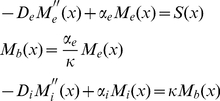
(2)where we introduced an “external effective degradation constant” 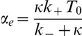 which corresponds to a linear binding rate. The external, bound and internalized components are then obtained by first solving analytically the Ordinary Differential Equation (ODE) for *M_e_*(*x*), which is proportional to *M_b_*(*x*), and then the ODE for *M_i_*(*x*) involving the effective source term *κM_b_*(*x*). Thus the equations in (2) can be transformed analytically into integral form and solved explicitly for the stepwise constant function *S*(*x*) (cf. Dalessi et al., in preparation and Text S1), whereas the general problem (1) can only be solved numerically.

**Parametrical study**

To the best of our knowledge, most of the parameters *D_e_*, *D_i_*, *α_e_*, *α*_i_, and *κ* have not yet been measured experimentally. We however aimed to constrain the parameter space using the available experimental observations and making some reasonable assumptions. We first notice that the knowledge of the relative abundance of each Dpp component (external, Tkv-bound and internalized) imposes a unique value of the *α_e_*, *κ*, and *α*_i_ parameters. The remaining diffusive parameters, namely *D_e_* and *D_i_*, can be fixed assuming that the total Dpp profile 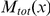 decays exponentially outside the Dpp production region with decay length 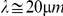 [26] (see also the exponential fit in Figure S1). Finally, in order to ensure that every component displays a biologically meaningful profile (i.e. negligible Dpp levels at the pouch boundaries), the internal diffusion constant *D_i_* can range only from 0 to a maximal limiting value 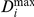. We studied three extreme limiting scenarios corresponding to the total Dpp being mainly external (80% of *M_tot_*(*x*) is external, 10% is Tkv-bound, and 10% is internalized), mainly Tkv-bound or mainly internalized.

**Modeling clone effects**

In *tkv* mutant clones, the total number of receptors *T_0_* is affected. In our model, we consider 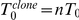 inside the clone with *n* = 0 for LOF experiments and n>1 in the GOF case. Transcytosis is receptor-mediated and therefore also affected by the presence of the clone. We assume that the effective internal diffusion constant depends linearly on the receptor number, yielding 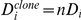. We obtain an analytical expression for the Dpp profile by solving the differential equations separately outside and inside the clone and connecting the solutions at the clone boundaries (cf. Text S1). The LOF and GOF *tkv* clone profiles related to the three different scenarios (cf. Figure 1), are obtained using the corresponding parameters and assuming a constant morphogen production rate over a small finite region (15% of the half wing pouch length *L*). For the scenario where Dpp is mainly internalized, we set the internal diffusion to its maximal allowed value (RMT model). For the other two cases, in which Dpp is mainly external or mainly receptor-bound, the gradient formation is dominated by the external diffusion, and transcytosis can therefore be neglected (RED models).

We then modeled the effects of clones containing either a 10-fold increase of receptor levels (gain-of-function, GOF) or entirely lacking the receptors (loss-of-function, LOF) on the Dpp gradient in the three different scenarios. The computed Dpp profiles are represented in Figure 1. All three transport scenarios predict for the GOF clones an increase of Dpp within the clone territory. Thus the GOF situations are not suited to discriminate between the RMT and RED models, but they can be exploited to test in vivo which of the Dpp signaling receptors, the type I receptor Tkv or the type II receptor Punt, binds to Dpp. LOF clones, however, clearly lead to qualitatively different Dpp profiles for each transport scenario. Most importantly, Dpp levels *behind* LOF clones are decreased in the RMT model, but are almost unchanged in the RED models (Figure 1D to 1F). This outcome reflects the necessity of receptors in transporting Dpp by RMT. Consistent with this, we find that in a scenario in which the majority of Dpp is intracellular, but Dpp is only transported by extracellular diffusion (the term describing transcytosis is set to zero), Dpp levels behind clones are almost unchanged (Text S1, Section 5.4). Thus, analyzing Dpp levels behind receptor mutant clones allows us to clearly discriminate between receptor-mediated transcytosis and restricted extracellular diffusion scenarios. Moreover, analyzing Dpp levels within receptor LOF clones allows one to discriminate between the limit case scenarios in which Dpp is either mainly “receptor-bound” or mainly “external-unbound,” and quantifying the Dpp levels inside GOF clones allows us to further narrow down the ratio of receptor-bound versus unbound Dpp.

### Genetic Tools for Studying the Dpp Gradient Across Receptor Mutant Clones

**Figure 2:**
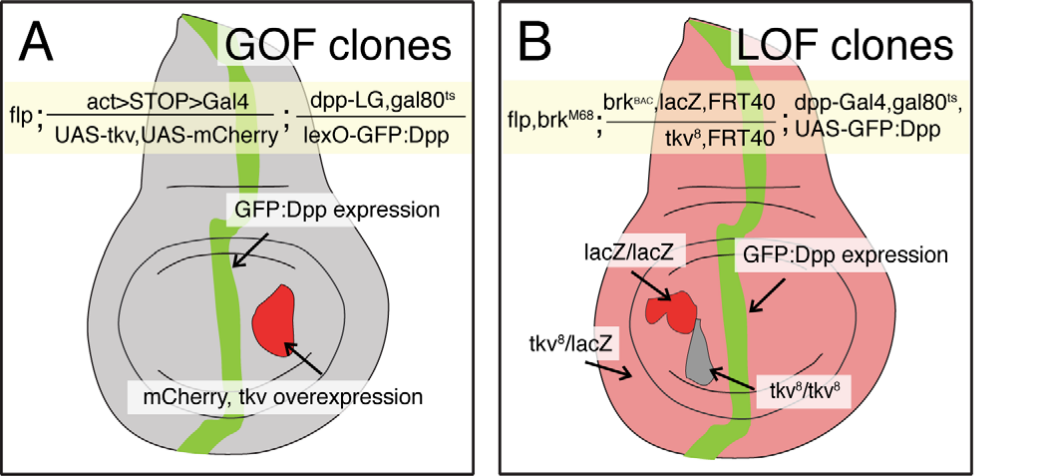
Analysis of the Dpp gradient in GOF and LOF receptor mutant clones. Sketches of 3^rd^ instar wing imaginal discs containing GOF (A) and LOF (B) receptor mutant clones. In both cases the Dpp gradient is visualized by expressing a GFP:Dpp fusion protein within its endogenous domain. In the GOF situation, we used the Lex-A binary expression system to express GFP:Dpp, and in the LOF situation we used the Gal4 binary expression system. Genotypes are described in the yellow boxes and in the Materials and Methods section. GOF clones are marked by co-expression of *mCherry*; LOF clones are marked by the loss of *arm-lacZ* or *ubi-nGFP* expression. In the clonal experiments where we analyzed the endogenous Dpp gradient by pMad or Sal stainings, GFP:Dpp was not expressed.

The experimental analysis of the Dpp gradient is complicated by the lack of antibodies that detect the mature, processed form of Dpp. Visualization, however, can be achieved using a GFP-tagged version of Dpp [5],[9]. Because the expression of GFP:Dpp in the *dpp* expression domain requires the Gal4 system, the generation of receptor GOF clones posed a problem. We therefore developed LexA-based transgenes that allowed to express GFP:Dpp with a Gal4-independent binary expression system [36], and employed an *actin5c>stop>Gal4* flp-out construct to generate and mark clones overexpressing the Dpp receptor (Figure 2A). The LexA-based GFP:Dpp gradient resembles the Gal4-based GFP:Dpp gradient (Figure S1), which has been shown to coincide with the endogenous Dpp activity gradient [5],[9],[28].

The analysis of the receptor LOF clones is hindered by the role of Dpp as a survival and growth factor. Cells within the wing primordium that lack Dpp signaling activity are efficiently eliminated, in particular when they are located close to the source where Dpp levels are normally high [21],[37]–[42]. This elimination is caused by the upregulation of Brk in Dpp signaling mutant cells. Hence we sought to prevent this response by genetically generating cells that not only lose Dpp receptor activity but simultaneously also *brk* function. However, since the genes encoding Dpp receptors and Brk are located on different chromosome arms, we combined BAC-recombineering and the phiC31 site-specific integration system [43]–[45] to position a genomic *brk* rescue construct at chromosomal site 22A, on the same chromosome arm where the type I Dpp receptor *tkv* is located. Mitotic recombination at the base of this chromosome arm in a *brk* mutant background enabled us to generate *tkv brk* double mutant clones (for details see Figure 2B).

### Effects of Receptor GOF Clones on the Dpp Profile

**Figure 3:**
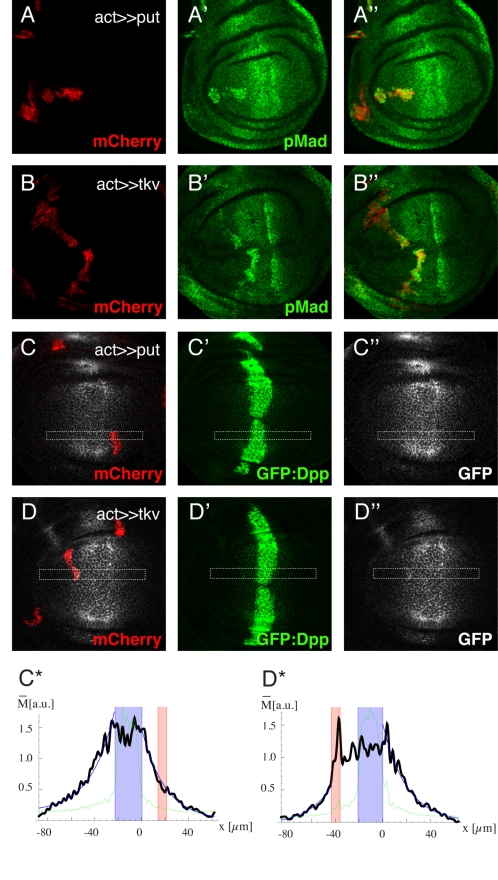
Receptor GOF studies reveal that Dpp binds to Tkv but not Punt. (A–D) Third instar wing imaginal discs containing *punt* (A,C) or *tkv* (B,D) overexpression clones. Clones are visualized by *mCherry* co-expression (A–D). pMad stainings (A′,B′) show that overexpression of *tkv* as well as *punt* leads to an increase in Dpp signaling activity, demonstrating the functionality of both constructs. However, only *tkv* but not *punt* overexpression leads to an increase in GFP:Dpp levels inside the clones (C,D). GFP:Dpp is visualized directly (C′,D′) and by antibody staining (C″,D″). (C*,D*) Intensity plots of the marked regions of the corresponding immunofluorescence images. The green line represents the GFP:Dpp signal, and the black line represents the intensities of the GFP antibody staining. The Dpp production region is indicated in blue, and the clone region in red. The position x is expressed in µm and the extracted Dpp levels in arbitrary units.

The Dpp ligand signals through the Tkv-Punt typeI-typeII receptor complex. Upon ligand-receptor binding, Tkv becomes phosphorylated at a glycine/serine rich domain, and in turn phosphorylates and activates Mad [14],[46]. While both receptors are necessary for the signal relay, in vitro studies suggest that Dpp binds to Tkv with high affinity, but not to Punt [14],[17],[34]. Here, we reassess these observations in vivo, by analyzing the effect of *tkv* and *punt* overexpression on Dpp distribution. As mentioned before, our theoretical clonal study predicts that any increase in receptor levels will also lead to increased Dpp levels, irrespective of the transport model (Figure 1A to 1C). To confirm the functionality of the transgenes, we first assessed the levels of Tkv by use of an antibody and estimated that the *UAS-tkv* transgene results in an approximately 10-fold increase at the protein level (Figure S2). We then verified that overexpression of *tkv* as well as *punt* ectopically activates Dpp pathway activity by monitoring the phosphorylation state of Mad (pMad) (Figure 3A and 3B). Finally, we analyzed the effect of *tkv* and *put* overexpression on the Dpp gradient. Throughout this work we monitor the Dpp gradient by directly measuring the GFP:Dpp fluorescence intensities (in green) and by GFP antibody staining (in gray). In order to avoid detection of unsecreted Dpp in producing cells and elution of GFP:Dpp from the ECM during fixation, we added the GFP antibody prior to fixation, followed by a 1-hour incubation at room temperature (for details, see Materials and Methods). Strikingly, only *tkv* overexpressing clones but not *punt* overexpressing clones modulate the Dpp profile and lead to an increase of GFP:Dpp levels inside the clones (Figure 3C and 3D; additional plots for each genotype are shown in Figures S3 and S4). Thus the comparison of the effect of *tkv* versus *punt* overexpression clones on the Dpp profile confirms biochemical studies and suggests that Tkv, but not Punt, binds to Dpp. Moreover, because the amplification of Dpp signal transduction per se (also occurred in UAS-punt clones) does not influence the Dpp profile, we can exclude the possibility that the observed effects in Tkv GOF clones are indirect, and argue that they are a direct consequence of Dpp-Tkv binding. Although the GOF studies do not enable distinguishing between the RMT and RED models, the different amounts of Dpp in GOF clones can serve to discriminate between the two RED scenarios. Our data favor the “external-unbound limit case scenario” and suggest that approximately 60%–80% of Dpp is not bound to Tkv (Figure S4G).

### Effects of Receptor LOF Clones on the Dpp Profile

**Figure 4:**
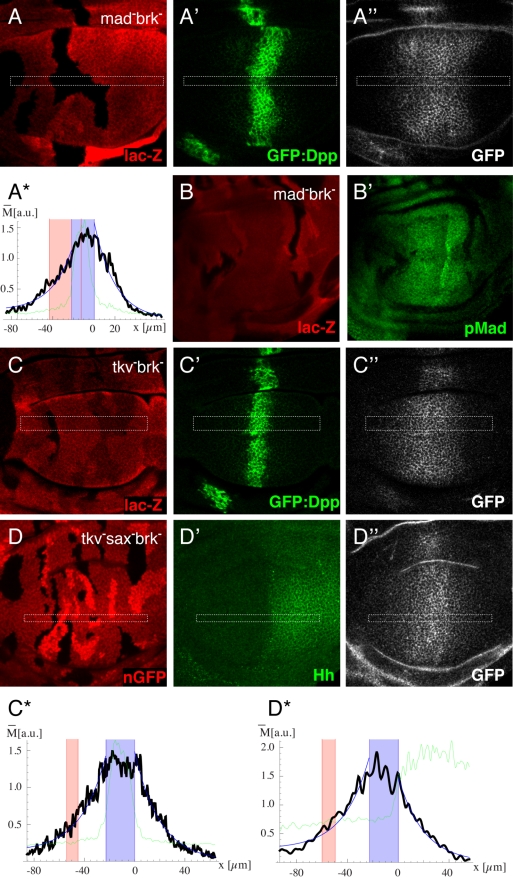
Effect of receptor LOF clones on the Dpp gradient is consistent with the restricted diffusion model. Third instar wing imaginal discs with LOF clones for *Mad brk* (A,B), *tkv brk* (C), and *tkv brk* in a *sax* mutant background (D). Receptor mutant clones are indicated by the loss of lac-Z staining (A,B,C) or nGFP (D). (B) Analysis of Dpp signaling activity by pMad staining (B′). (A,C,D) Analysis of the Dpp gradient. GFP:Dpp is visualized directly (A′,C′) and by antibody staining (A″, C″, D″). While GFP antibody staining in (D) did not interfere with the nuclear GFP clone-marker (see Materials and Methods), direct visualization of GFP:Dpp was not possible in this genotype. In order to estimate the region of Dpp production in this genotype, we visualized the A-P boundary by Hh antibody staining (D′). (A*,C*,D*) Intensity plots of the marked regions of the corresponding immunofluorescence images. The green line represents the GFP:Dpp (A,C) or Hh (D) signal, and the black line represents the intensities of the GFP antibody staining. The Dpp production region is indicated in blue, and the clone region in red. The position x is expressed in µm and the extracted Dpp levels in arbitrary units. None of the genotypes lead to a significant effect on the Dpp gradient. Therefore the data contradict the RMT scenario and coincide with the RED scenario in which the majority of Dpp is external-unbound.

As described above, receptor LOF situations were created by simultaneous removal of the receptor and *brk*. First we tested whether the alteration of Dpp signaling activity in such clones (loss of Dpp transduction and loss of *brk* function) would affect the Dpp profile, and generated *Mad*, *brk* double mutant clones, in which Dpp transduction but not receptor activity is lost. The Dpp gradient across such clones remains intact (Figure 4A,B—additional plots are shown in Figure S5). As a consequence of epithelial folds that occasionally arise at the boundaries of such clones, in some cases a slight modulation of the Dpp profile was observed (Figure S5).

**Figure 5:**
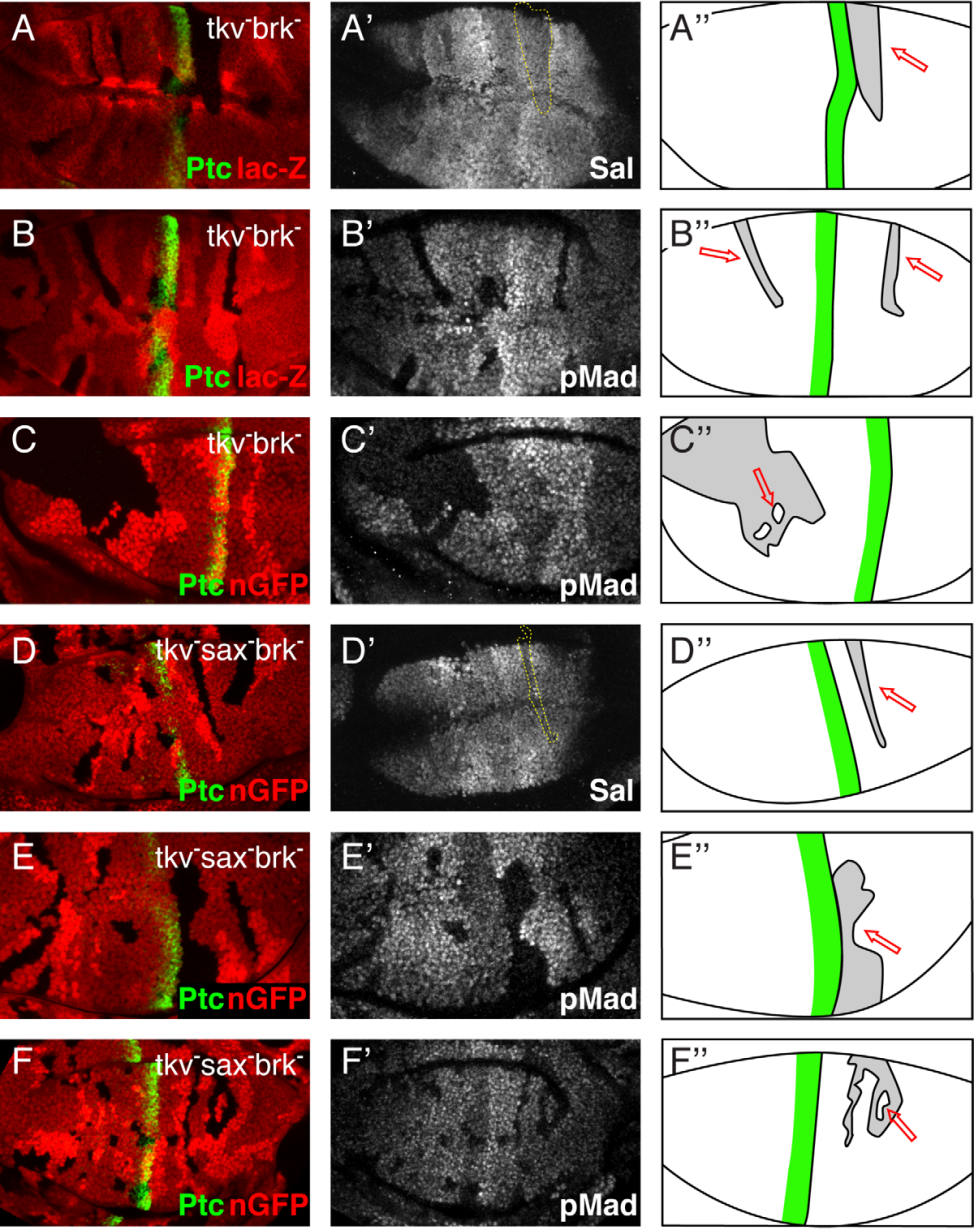
Movement of endogenous Dpp through receptor mutant clones refutes the receptor-mediated transcytosis model. Third instar wing imaginal discs with LOF clones for *tkv brk* (A–C) and *tkv brk* in a *sax* mutant background (D–F). (A–F) The Dpp production region is indicated by staining for the Hh target Patched (green), and receptor mutant clones are shown by the loss of *lac-Z* expression (A,B) or nGFP (C–F). The Dpp gradient is visualized indirectly, by staining for the pathway activity (pMad) (B′,C′,E′,F′), or by staining for the Dpp target gene *spalt* (A′,D′). (A″–F″) Sketches of the analyzed wing discs. The green line indicates the Dpp production region, considered clones are shown in gray, and red arrows indicate the regions behind clones in which high Dpp signaling activity can only occur if Dpp moves through receptor mutant tissue, which is indeed the case, as high Spalt and pMad signals can be observed in these regions.

We then examined the Dpp gradient in discs with *tkv*, *brk* LOF clones. We used the amorphic *tkv^8^* allele, which contains a stop mutation in the extracellular domain of *tkv* at position 144 of the *tkv-PA* transcript [47]. As expected, Dpp signal transduction activity was abolished in *tkv* ^−^ *brk* ^−^ clones (Figure 5B–C). However, the Dpp gradient in such discs was not significantly altered; *tkv* ^−^ *brk* ^−^ clones resembled *Mad* ^−^ *brk* ^−^ clones (Figure 4C—additional plots are shown in Figure S6). The same results were obtained using a conventional antibody staining protocol to detect the Dpp gradient (Figure S8). The observation that the Dpp levels inside and behind *tkv* ^−^ clones are not significantly reduced contradicts the receptor-mediated transcytosis model, and concurs with the restricted extracellular diffusion model, in which the majority of Dpp is not bound to Tkv (see Figure 1).

Apart from Tkv the Drosophila genome encodes another type I receptor, Saxophone (Sax), which has been implicated in Dpp signaling. Although Sax preferentially interacts with and mediates signaling by the BMP ligand Glass Bottom Boat (Gbb) and shows significantly lower affinity to Dpp than Tkv [10],[34],[48],[49], it could still in principle serve as a Dpp receptor. To exclude that Sax takes over some functions of Tkv in *tkv brk* mutant clones, for example shuttling Dpp through mutant cells via receptor-mediated transcytosis, we also analyzed the Dpp gradient in *sax* null discs containing *tkv* ^−^ *brk* ^−^ clones (see Materials and Methods). GFP:Dpp levels were not decreased, neither inside nor behind the clones, strengthening our conclusions that GFP:Dpp does not move via receptor-mediated transcytosis (Figure 4D—additional plots are shown in Figure S7).

Finally we also analyzed the effect of receptor LOF clones on the endogenous Dpp gradient in wing discs. We tested *tkv* ^−^ *brk* ^−^ and *tkv* ^−^ *brk* ^−^ *sax* ^−^ genotypes and monitored Dpp pathway activity at the level of Mad phosphorylation and target gene expression. Since Dpp could potentially reach the distal side of such clones by being transported around, rather than through, mutant territory, we purposely collected and analyzed clones with a large dorsoventral extension. Both readouts, however, show that Dpp signaling is not reduced behind such receptor mutant clones (Figure 5A–C). To completely eliminate the possibility that this pathway activity stems from Dpp that migrated via clone-surrounding wild-type cells, we identified rare situations where patches of wild-type cells are fully encircled by mutant cells. As shown in Figure 5C and 5F, even cells in these “islands” exhibit substantial Dpp signaling activity (for a 3-D reconstruction of these clone islands, see Figure S9). These findings provide unequivocal evidence that Dpp can disperse through receptor-free territory and hence refute a need for receptor-mediated transcytosis.

## Discussion

Dpp acts as a long-range morphogen, which spreads along the A-P axis of the wing primordium to form a signaling gradient. Here we studied how receptor mutant clones affect the Dpp gradient in different transport models, and compared theoretical calculations with experimental data.

One outcome of the modeling was the prediction that RMT and RED mechanisms could be discriminated by analyzing Dpp levels behind receptor mutant clones. While in the transcytosis model these levels should be significantly decreased, they would be almost unaltered in the diffusion model. This difference stems from the uptake of Dpp by its receptors, which is an essential feature for morphogen transport by RMT, but not by RED. Our experimental results revealed that neither GFP:Dpp levels nor Dpp signaling activity is reduced behind receptor mutant clones, excluding a significant role for receptor-mediated transcytosis in Dpp gradient formation. Important support for this conclusion was provided by situations where “islands” of wild-type cells received Dpp signal despite being surrounded by mutant tissue, ruling out the possibility that Dpp reaches the distal side of receptor mutant clones by being transported around the clones. When analyzing the GFP:Dpp distribution in mosaic tissues, we also found that the Dpp levels are not significantly reduced within receptor mutant clones. While this outcome further argues against the RMT model, it is consistent with the “external-unbound limit case scenario,” representing RED with the majority of Dpp not being bound to Tkv. Indeed, in the GOF experiments the ratio of unbound Dpp could be narrowed down to approximately 60%–80%.

If transcytosis is modeled in a receptor-*independent* manner (as shown in Text S1), the effects on Dpp distribution by receptor mutant clones do not differ significantly from those in the restricted extracellular diffusion scenario. Thus, receptor-independent transcytosis, for example via fluid phase uptake, remains a possible mechanism for Dpp gradient formation. Several other studies, however, support the restricted extracellular diffusion model. Based on theoretical grounds, Lander et al. (2002) [27] proposed that diffusive mechanisms for Dpp gradient formation are more likely than non-diffusive ones. Moreover, experimental studies on heparan sulfate proteoglycans (HSPGs), in particular glypicans, demonstrated the necessity of an intact ECM for morphogen movement [50],[51]. In the Drosophila wing disc, clones mutant for the glypicans Dally and Dally-like (Dlp) disrupted the formation of the Dpp gradient [28]. Dally was also shown to bind Dpp [52], to stabilize it on the cell surface [53], and to influence its mobility [54],[55].

However, although the evidence that glypicans assist extracellular diffusion of Dpp seems compelling, alternative or additional functions of glypicans in Dpp distribution cannot be excluded. For example, a recent study [56] suggests that apically localized Dlp binds to the Wingless (Wg) morphogen in the Wg producing region, undergoes internalization, and thereby redistributes Wg to the basolateral compartment where Wg spreads to form a long-range gradient. It is possible that recycling of glypicans is also involved in Dpp relocalization and that this process is important for Dpp movement. Consistent with such a notion, Kicheva et al. (2007) [26] reported that dynamin-dependent endocytosis is necessary for Dpp movement. Blocking such a ubiquitous cellular machinery, however, not only inhibits the recycling of receptors and glypicans, but may also change the composition and distribution of glypicans in the ECM, which in turn might impede extracellular diffusion. Given that the phenotypes of our receptor clones fully conform to the simplest model of Dpp movement along the ECM (restricted extracellular diffusion), we favor the view that the main function of glypicans for Dpp gradient formation is to facilitate Dpp diffusion along the ECM.

Our observation that receptor mutant clones do not have a major effect on the Dpp gradient contradicts previous observations by Entchev et al. (2000) [5]. In their study, ablation of *tkv* in small lateral clones leads to an accumulation of Dpp at the side of the clone facing the source, arguing for a block of Dpp movement within such clones [5]. The different results could be explained by the presence of *brk* in their genetic setup. The ectopic up-regulation of *brk* in *tkv* mutant clones, which in most cases leads to clone elimination [41],[42], most likely also causes drastic changes in the transcriptional program in “escaper” cells. Thus the sharp increase in GFP:Dpp levels at the proximal edge inside *tkv* mutant clones (facing the Dpp source) could be accounted for by increased levels of Dpp binding proteins, a theory which is supported by the fact that Dpp accumulation was strictly clone-autonomous and not in cells ahead of the clones [27]. In our experimental setup, we avoided such secondary effects by simultaneously removing *tkv* together with *brk*. As our negative control (*Mad brk* clones) shows, the signaling state of these cells (Dpp signaling off, no Brk) does not significantly alter the Dpp profile.

Transport along cytonemes is another proposed model for the dispersal of Dpp (Ramirez-Weber and Kornberg, 1999) [33]. In its simplest implementation, this model assumes that imaginal disc cells form filopodial extensions towards the Dpp producing region and that Dpp is shuttled along these extensions by binding to Tkv [32]. In this scenario, Tkv GOF clones would not only lead to an increase of receptors inside the clones, but also along the cytonemes, and thus affect the Dpp profile also ahead of the clones. This, however, was not observed in our experiments (Figure 3D and Figure S4), and we therefore favor the restricted extracellular diffusion model over the cytoneme model for Dpp gradient formation.

During development morphogens function as short-range or long-range signals in order to specify cell fates within a tissue. For example, during wing disc development the range of Hh signaling is relatively short compared to that of Dpp, with a functional range of approximately 10 cells versus 40 cells, respectively [7],[9],[57],[58]. It is likely that properties of the transport system are important determinants of the range of a morphogen. In the restricted diffusion model, morphogen spreading is impeded by ECM proteins and cell surface receptors, which efficiently trap their ligand at the cell surface and direct it to degradation. Thus one mechanism to control the range of a morphogen gradient is regulating the receptor levels [27]. Indeed, the Hh as well as the Dpp system appear to make use of this strategy to regulate their range. The Hh signal limits its range by upregulating the expression of its binding receptor Patched (Ptc), while the Dpp signal broadens its range by downregulating the expression of its receptor Tkv [6],[57],[59]. The effects of our Tkv LOF and GOF clones on the Dpp profile suggest that the majority of Dpp is not bound to the receptor Tkv. It is tempting to speculate that the Dpp-Tkv binding properties represent an additional property of the Dpp signaling system that facilitates the formation of a long-range gradient, by assuring that the majority of Dpp remains in a free and unbound state. Just like lower receptor levels, a lower binding constant would contribute to the spread of Dpp, due to reduced immobilization and degradation of Dpp. It remains to be seen if the ratio of bound to unbound ligand differs for long- versus short-range morphogens and if this ratio represents a general means to regulate the range of morphogen gradients.

## Materials and Methods

### Fly Lines

The following transgenes and mutants are described in detail on *flybase*: *UAS-mCherry-CAAX*, *tub-Gal80^ts^*, *hsp70-flp*, *act5C>y+>Gal4*, *arm-lacZ*, *ubi-GFP(S65T)nls*, *brk^M68^*, *tkv^8^*, *sax^P^*, *mad^B1^*, and *FRT40*. Furthermore we used the transgenes: *UAS-tkv* [59], *UAS-punt* [7], *lexO-GFP:Dpp* [36], *dpp-LG* [36], *dpp-Gal4* [5], and *UAS-GFP:Dpp* [5].

### Clone Induction

#### LOF clones

Crosses were kept at 18°C. Clones were induced 96 h after egg laying (AEL), and then kept for another 96 h at 18°C. Then larvae were shifted for 16 h to 29°C (permissive temperature for the Gal80^ts^ system), which allows the formation of a steady state GFP:Dpp gradient. Only male larvae were *brk* mutant and thus picked.

#### GOF clones

Crosses were again kept at 18°C. Clones were induced 144 h AEL, kept for 24 h more at 18°C, and then shifted for 24 h to 29°C in order to allow expression of the UAS-transgenes and *lexO-GFP:Dpp*.

### BAC-Recombineering

In order to introduce the manipulated *brk* locus into the fly genome, the locus was transferred from the original BAC (BACR35J16) into the attB-P[acman] vector, which allows the retrieval of large fragments up to 133 kb (Venken et al., 2006) [44]. The BAC clone was ordered from BACPAC Resources, and the BAC DNA isolated according to the protocol provided. Homology arms of 500 bp, corresponding to the 5′ and 3′ ends of the entire *brk* genomic locus and spanning parts of the upstream unc-119 gene and the downstream Atg5 gene, were cloned into the attB-P[acman] vector. The attB-P[acman] vector was linearized and introduced into recombination-competent *SW102* carrying the modified BAC. The retrieval of the modified DNA fragment into the linearized attB-P[acman] was carried out by recombination-mediated gap-repair. This plasmid was then injected into *Drosophila melanogaster* embryos. Site-specific integration of the attB-P[acman] vector into the landing site 51D on chromosome 2L was performed as described [43].

### Immunohistochemistry

Immunostainings were performed using standard protocols. Images were collected with a Zeiss LSM710 confocal microscope. ImageJ was used to analyze the images; z-stacks are shown in maximum projections. Intensity plots were generated based on the extraction of the intensities in the ROI's and using Mathematica. For the 3-D reconstruction of z-stacks, Imaris was used.

#### GFP antibody staining protocol

The extracellular environment greatly reduces the GFP fluorescence compared to the intracellular environment, and extracellular GFP:Dpp is washed out of the extracellular matrix. In order to also stain for the external GFP:Dpp pool, we developed the following protocol: Wing discs were dissected in Clone8 medium and GFP antibody was added to a final dilution of 1∶50. After 1 h of incubation at RT, discs were washed 3 times with PBS, fixed for 15 min without detergent, and then for 10 min adding Triton X-100. This way the GFP-antibody was able to bind to GFP:Dpp in vivo, and in the 1 h incubation time GFP:Dpp could also be internalized by cells. The extracellular GFP:Dpp pool was not washed out using this protocol; thus this protocol represents all three pools of Dpp: the extracellular, receptor-bound, and internalized Dpp. After the fixation step, standard protocols were followed for additional antibody stainings and the secondary antibody stainings.

The following antibodies were used: mouse anti-ß-Gal (Promega), rabbit anti-pMad (gift from Ed Laufer, Columbia University, New York), mouse anti-Patched (Hybridoma bank), rabbit anti-Sal (gift from Ronald Kuhnlein, Max-Planck-Institute, Göttingen), mouse anti-GFP (Millipore MAB3580), and rabbit anti-Tkv (Michael O'Connor, University of Minnesota). Secondary antibodies: Alexa Flour antibodies (Molecular Probes).

## Supporting Information

Figure S1 Analysis of the GFP:Dpp gradient in wild-type discs. Expression of *UAS-GFP:Dpp* under the control of *dpp-Gal4* driver in 3^rd^ instar wing imaginal discs. GFP:Dpp is visualized directly (A) and by antibody staining (A′). Expression of *lexO-GFP:Dpp* under the control of *dpp-LG*, direct visualization in (B), and by antibody staining in (B′). (A*,B*) Intensity plots of the marked regions of the corresponding immunofluorescence images. The green line represents the GFP:Dpp signal, and the black line represents the intensities of the GFP antibody staining. The Dpp production region is indicated in blue. The thin blue line represents the exponential fits to the Dpp profile outside of the production region. A decay length of 20%, used as a parameter for our modeling, is a good approximation to the wild type Dpp gradient. The positions on x are expressed in µm and the extracted Dpp levels in arbitrary units.
(PDF)

Figure S2 Analysis of the Tkv levels in the Tkv-GOF clones. Expression of *tkv* in 3^rd^ instar wing imaginal discs under the control of the *actin5c>stop>Gal4* flp-out construct. The immunofluorescence images show Tkv antibody staining in (A) and the UAS-mCherry clone marker in (A′). (B) Intensity plot of the Tkv antibody staining levels of the region marked in A and A′. Tkv levels indicate an approximately 10-fold increase of receptor levels inside the GOF clones compared to the wild-type levels in the surrounding tissue.
(PDF)

Figure S3 Effect of *punt* overexpression clones on the Dpp gradient. (A–E) Intensity plots of the Dpp profile from immunofluorescence images of 3^rd^ instar wing imaginal discs containing *punt* overexpression clones (for an example image, see Figure 3C). The green line represents the GFP:Dpp signal, and the black line represents the intensities of the GFP antibody staining. The Dpp production region is indicated in blue, and the clone region in red. The position x is expressed in µm and the extracted Dpp levels in arbitrary units. *punt* overexpression clones do not lead to a significant effect on the Dpp gradient, suggesting that the typeII receptor Punt does not bind to Dpp.
(PDF)

Figure S4 Effect of *tkv* overexpression clones on the Dpp gradient. (A–F) Intensity plots of the Dpp profile from immunofluorescence images of 3^rd^ instar wing imaginal discs containing *tkv* overexpression clones (for an example image, see Figure 3D). The green line represents the GFP:Dpp signal, and the black line represents the intensities of the GFP antibody staining. The Dpp production region is indicated in blue, and the clone region in red. The position x is expressed in µm and the extracted Dpp levels in arbitrary units. *tkv* overexpression clones lead to a significant increase of Dpp levels inside clones, suggesting that the typeI receptor Tkv binds to Dpp. (G) A strict distinction between the two RED scenarios is not possible, as they only differ in the ratio of Tkv-bound versus unbound Dpp. In order to determine this ratio, we quantified the increase of Dpp levels inside the *tkv* overexpressing clones shown in Figure 3D and Figure S4A–F, and calculated the ratio from these data (for a detailed description, see Text S1). The n-fold increase of receptor levels inside clones (*x*-axis) ranges from 0 to 20. The *y*-axis shows the ratio of free receptors (a = 1 corresponds to 100% of external unbound Dpp, a = 0 to 100% of external Tkv-bound Dpp). The blue lines show the values for *n* = 10.
(PDF)

Figure S5 Effect of *Mad* ^−^ *brk* ^−^ clones on the Dpp gradient. (A–E) Intensity plots of the Dpp profile from immunofluorescence images of 3^rd^ instar wing imaginal discs containing *Mad* ^−^ *brk* ^−^ clones (for an example image, see Figure 4A). The green line represents the GFP:Dpp signal, and the black line represents the intensities of the GFP antibody staining. The Dpp production region is indicated in blue, and the clone region in red. The position x is expressed in µm and the extracted Dpp levels in arbitrary units. *Mad* ^−^ *brk* ^−^ clones do not lead to major alterations of the Dpp gradient. However, in some cases the clones lead to epithelial folds at the clone boundary, which can lead to minor irregularities in the Dpp gradient.
(PDF)

Figure S6 Effect of *tkv* ^−^ *brk* ^−^ clones on the Dpp gradient. (A–E) Intensity plots of the Dpp profile from immunofluorescence images of 3^rd^ instar wing imaginal discs containing *tkv* ^−^ *brk* ^−*-*^ clones (for an example image, see Figure 4C). The green line represents the GFP:Dpp signal, and the black line represents the intensities of the GFP antibody staining. The Dpp production region is indicated in blue, and the clone region in red. The position x is expressed in µm and the extracted Dpp levels in arbitrary units. *tkv* ^−^ *brk* ^−^ clones do not lead to major alterations of the Dpp gradient. Minor irregularities in the Dpp gradient seen here were already observed in the negative control (Figure S4); thus, we can assume that the loss of *tkv* does not have any influence on the Dpp gradient.
(PDF)

Figure S7 Effect of *tkv* ^−^ *brk* ^−^ clones on the Dpp gradient in *sax* ^−^ wing discs. (A–E) Intensity plots of the Dpp profile from immunofluorescence images of *sax* mutant 3^rd^ instar wing imaginal discs containing *tkv* ^−^ *brk* ^−^ clones (for an example image, see Figure 4D). The green line represents the GFP:Dpp signal, and the black line represents the intensities of the GFP antibody staining. The Dpp production region is indicated in blue, and the clone region in red. The position x is expressed in µm and the extracted Dpp levels in arbitrary units. *sax* ^−^ *tkv* ^−^ *brk* ^−^ cells do not lead to major alterations of the Dpp gradient. Minor irregularities in the Dpp gradient were already observed in the negative control (Figure S4); thus we can assume that the loss of the two type I receptors *sax* and *tkv* does not have any influence on the Dpp gradient.
(PDF)

Figure S8 Effect of *tkv* ^−^ *brk* ^−^ clones on the Dpp gradient using a conventional antibody staining protocol. Throughout the article we always analyzed the Dpp gradient using a special antibody staining protocol in order to preserve the extracellular GFP:Dpp pool (see main text and Materials and Methods). In this figure, we examined the effect of *tkv* ^−^ *brk* ^−^ clones on the Dpp gradient using a conventional antibody staining protocol. Confirming the results shown in Figures 4C and S6, also when using the conventional protocol, *tkv* ^−^ *brk* ^−^ clones did not alter the GFP:Dpp gradient. (A, A′) A 3^rd^ instar wing imaginal disc containing *tkv* ^−^ *brk* ^−^ clones. Receptor mutant clones are shown by the loss of lac-Z staining (A), and the GFP:Dpp gradient is visualized using a conventional antibody staining protocol (A′). (A*) GFP:Dpp intensity plot of the marked region of the immunofluorescence image. (B–E) More intensity plots of Dpp profiles of discs containing *tkv* ^−^ *brk* ^−^ clones using conventional antibody staining.
(PDF)

Figure S9 3-D reconstruction of wing imaginal discs containing clone “islands.” In Figure 5C and 5F, we show patches of wild-type cells fully encircled by *tkv* ^−^ *brk* ^−^ or *tkv* ^−^ *sax* ^−^ *brk* ^−^ mutant cells, which still exhibit substantial Dpp signaling activity (clone “islands”). The rotation of a 3-D reconstruction of the entire z-stack of these discs-images unambiguously shows that the wild-type clone “islands” are fully surrounded by mutant tissue in every z-plane. Dpp therefore has to pass through mutant tissue in order to reach the wild-type patches of cells. Here we show the 3-D reconstructions of the entire z-stack of the wing discs shown in Figure 5C (A) and Figure 5F (B) from one representative angle.
(PDF)

Text S1 Mathematical and theoretical details of the modeling presented in this article.
(PDF)

## Abbreviations

AEL: after egg laying
A-P: anteroposterior
Brk: Brinker
Dpp: Decapentaplegic
ECM: extracellular matrix
Gbb: Glass Bottom Boat
GOF: gain-of-function
HSPG: heparan sulfate proteoglycan
LOF: loss-of-function
Mad: Mothers-against-Dpp
pMad: phosphorylated form of Mad
Ptc: Patched
RED: restricted extracellular diffusion
RMT: receptor-mediated transcytosis
Sax: Saxophone
Tkv: Thick veins
Wg: Wingless
