# Supplementary figures and images for: Formation of the Long Range Dpp Morphogen Gradient
Source: PLoS Biol. 2011 Jul 26;9(7):e1001111. doi: 10.1371/journal.pbio.1001111 (PMC3144185; doi:10.1371/journal.pbio.1001111)

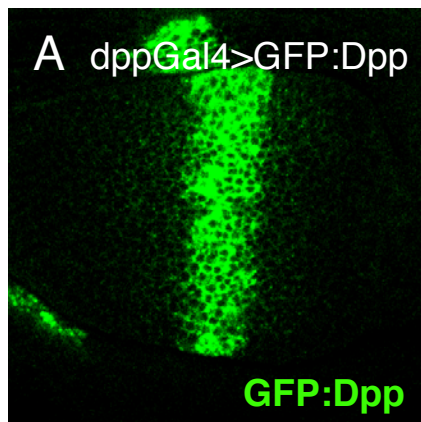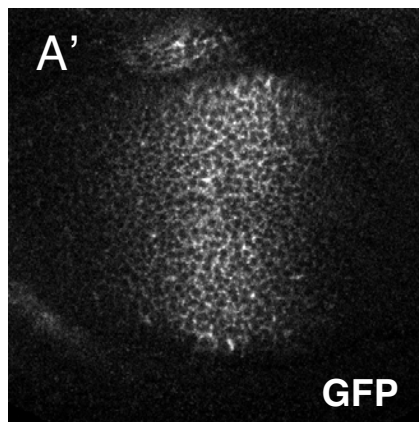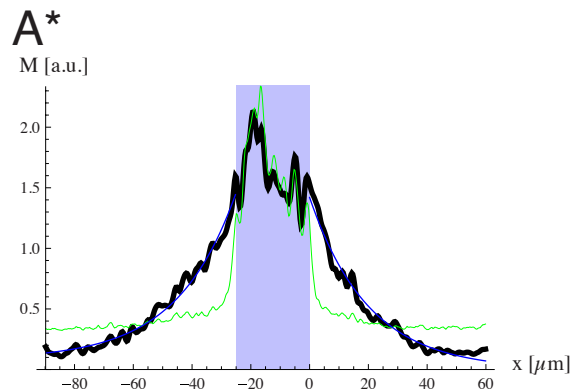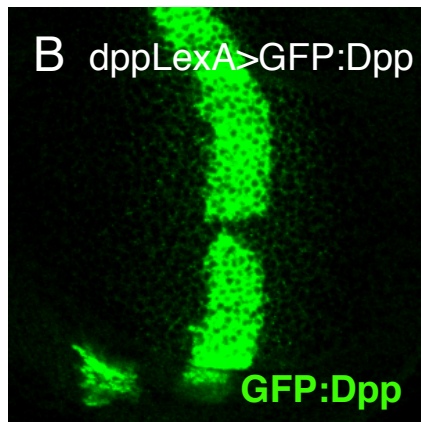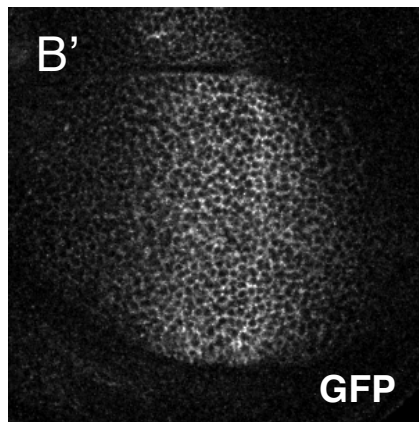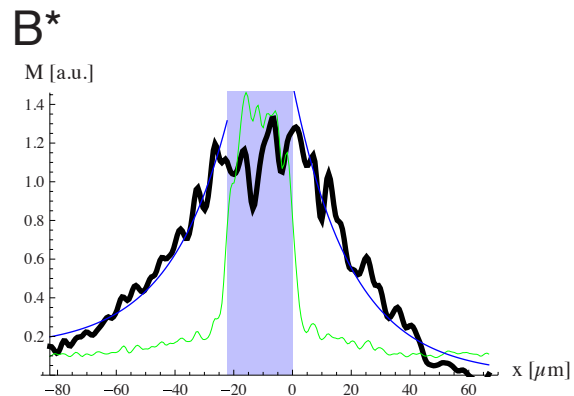

Supplement: Figure S1 — Analysis of the GFP:Dpp gradient in wild-type discs. Expression of UAS-GFP:Dpp under the control of dpp-Gal4 driver in 3rd instar wing imaginal discs. GFP:Dpp is visualized directly (A) and by antibody staining (A′). Expression of lexO-GFP:Dpp under the control of dpp-LG, direct visualization in (B), and by antibody staining in (B′). (A*,B*) Intensity plots of the marked regions of the corresponding immunofluorescence images. The green line represents the GFP:Dpp signal, and the black line represents the intensities of the GFP antibody staining. The Dpp production region is indicated in blue. The thin blue line represents the exponential fits to the Dpp profile outside of the production region. A decay length of 20%, used as a parameter for our modeling, is a good approximation to the wild type Dpp gradient. The positions on x are expressed in µm and the extracted Dpp levels in arbitrary units. (PDF) [file pbio.1001111.s001.pdf]

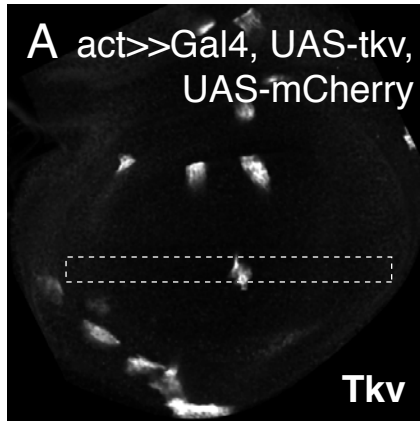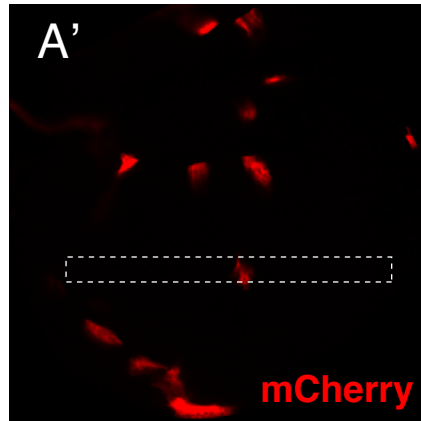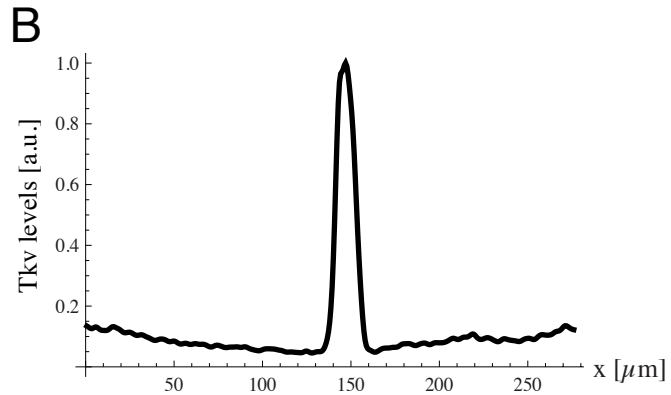

Supplement: Figure S2 — Analysis of the Tkv levels in the Tkv-GOF clones. Expression of tkv in 3rd instar wing imaginal discs under the control of the actin5c>stop>Gal4 flp-out construct. The immunofluorescence images show Tkv antibody staining in (A) and the UAS-mCherry clone marker in (A′). (B) Intensity plot of the Tkv antibody staining levels of the region marked in A and A′. Tkv levels indicate an approximately 10-fold increase of receptor levels inside the GOF clones compared to the wild-type levels in the surrounding tissue. (PDF) [file pbio.1001111.s002.pdf]

# *punt* GOF clones

## A

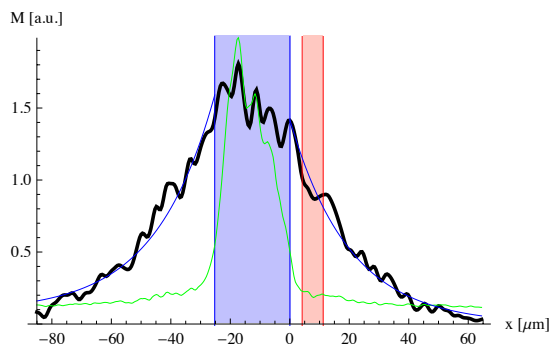

## B

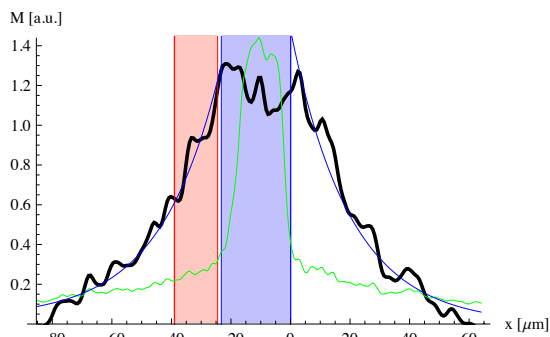

## C

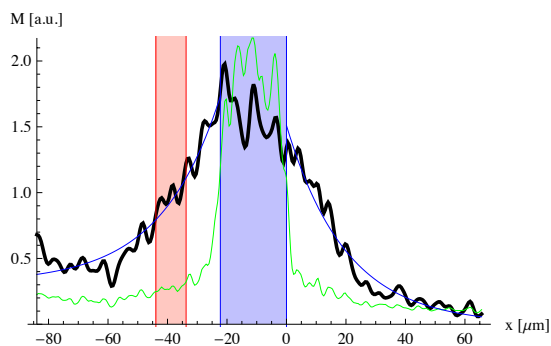

## D

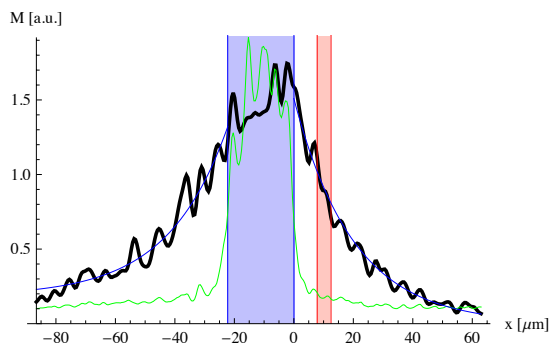

## E

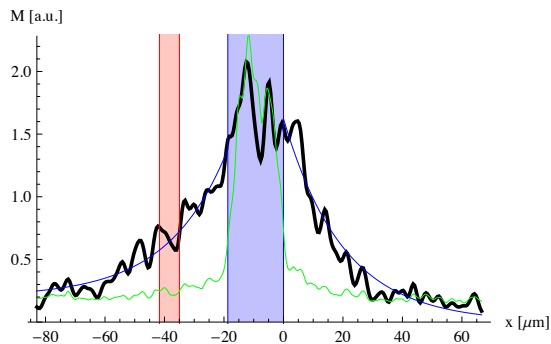

Supplement: Figure S3 — Effect of punt overexpression clones on the Dpp gradient. (A–E) Intensity plots of the Dpp profile from immunofluorescence images of 3rd instar wing imaginal discs containing punt overexpression clones (for an example image, see Figure 3C). The green line represents the GFP:Dpp signal, and the black line represents the intensities of the GFP antibody staining. The Dpp production region is indicated in blue, and the clone region in red. The position x is expressed in µm and the extracted Dpp levels in arbitrary units. punt overexpression clones do not lead to a significant effect on the Dpp gradient, suggesting that the typeII receptor Punt does not bind to Dpp. (PDF) [file pbio.1001111.s003.pdf]

# tkv GOF clones

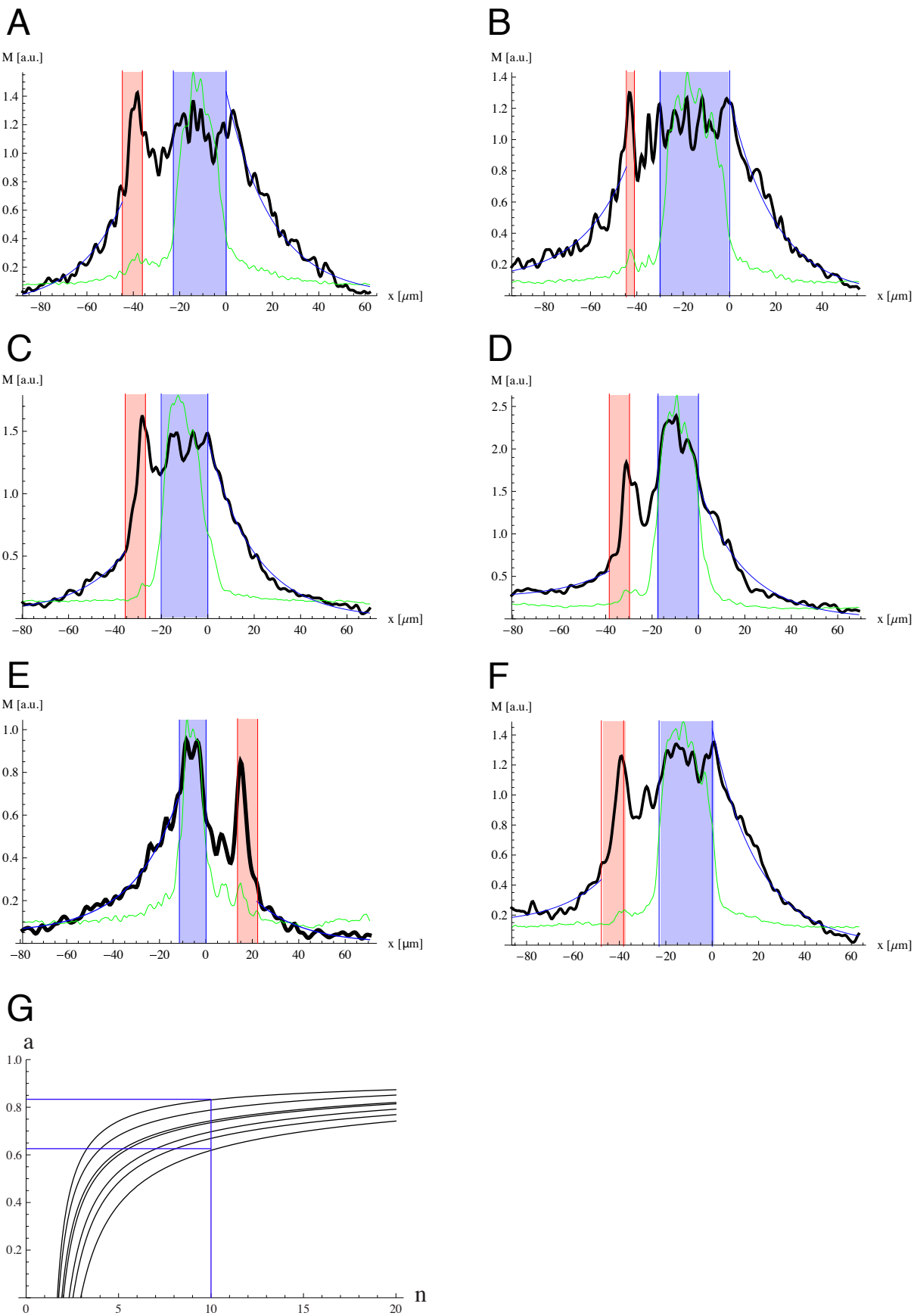

Supplement: Figure S4 — Effect of tkv overexpression clones on the Dpp gradient. (A–F) Intensity plots of the Dpp profile from immunofluorescence images of 3rd instar wing imaginal discs containing tkv overexpression clones (for an example image, see Figure 3D). The green line represents the GFP:Dpp signal, and the black line represents the intensities of the GFP antibody staining. The Dpp production region is indicated in blue, and the clone region in red. The position x is expressed in µm and the extracted Dpp levels in arbitrary units. tkv overexpression clones lead to a significant increase of Dpp levels inside clones, suggesting that the typeI receptor Tkv binds to Dpp. (G) A strict distinction between the two RED scenarios is not possible, as they only differ in the ratio of Tkv-bound versus unbound Dpp. In order to determine this ratio, we quantified the increase of Dpp levels inside the tkv overexpressing clones shown in Figure 3D and Figure S4A–F, and calculated the ratio from these data (for a detailed description, see Text S1). The n-fold increase of receptor levels inside clones (x-axis) ranges from 0 to 20. The y-axis shows the ratio of free receptors (a = 1 corresponds to 100% of external unbound Dpp, a = 0 to 100% of external Tkv-bound Dpp). The blue lines show the values for n = 10. (PDF) [file pbio.1001111.s004.pdf]

# *Mad*<sup>+</sup>*brk*<sup>-</sup> LOF clones

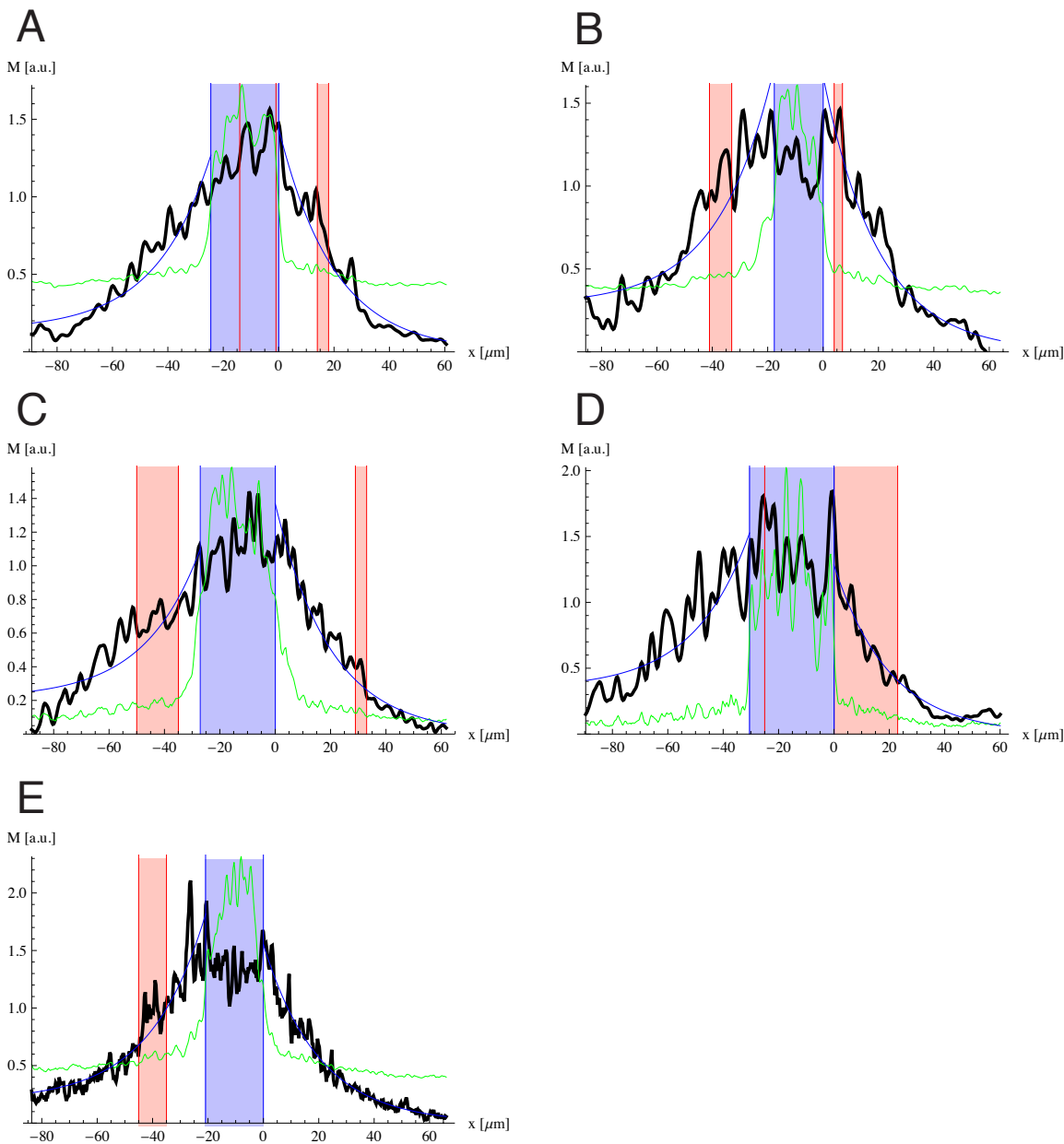

Supplement: Figure S5 — Effect of Mad − brk − clones on the Dpp gradient. (A–E) Intensity plots of the Dpp profile from immunofluorescence images of 3rd instar wing imaginal discs containing Mad − brk − clones (for an example image, see Figure 4A). The green line represents the GFP:Dpp signal, and the black line represents the intensities of the GFP antibody staining. The Dpp production region is indicated in blue, and the clone region in red. The position x is expressed in µm and the extracted Dpp levels in arbitrary units. Mad − brk − clones do not lead to major alterations of the Dpp gradient. However, in some cases the clones lead to epithelial folds at the clone boundary, which can lead to minor irregularities in the Dpp gradient. (PDF) [file pbio.1001111.s005.pdf]

# $tkv^{-}brk^{-}$ LOF clones

A

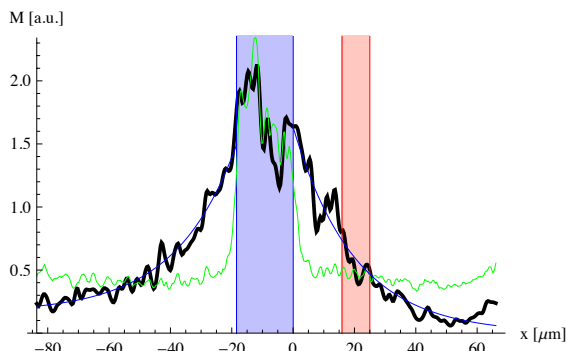

B

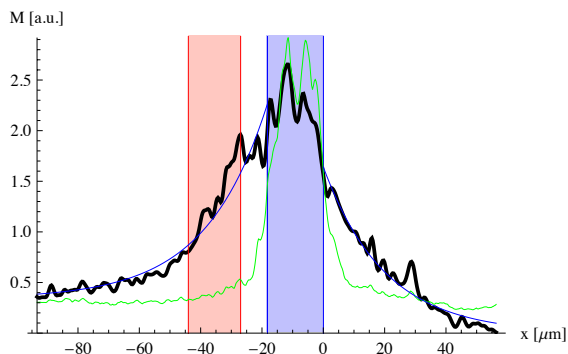

C

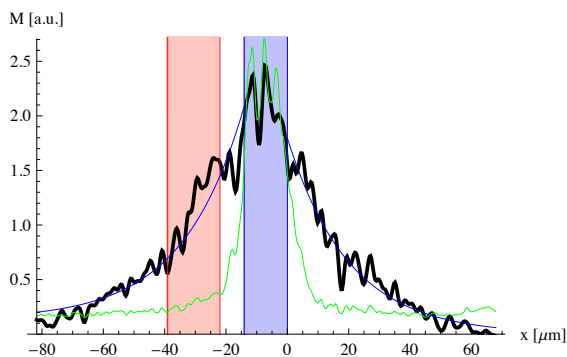

D

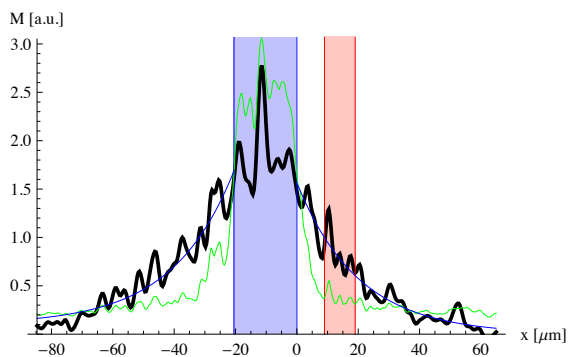

E

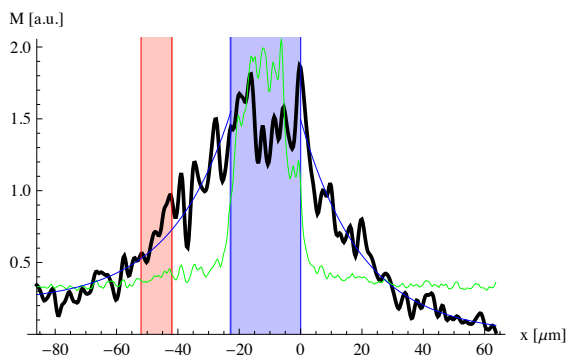

Supplement: Figure S6 — Effect of tkv − brk − clones on the Dpp gradient. (A–E) Intensity plots of the Dpp profile from immunofluorescence images of 3rd instar wing imaginal discs containing tkv − brk −- clones (for an example image, see Figure 4C). The green line represents the GFP:Dpp signal, and the black line represents the intensities of the GFP antibody staining. The Dpp production region is indicated in blue, and the clone region in red. The position x is expressed in µm and the extracted Dpp levels in arbitrary units. tkv − brk − clones do not lead to major alterations of the Dpp gradient. Minor irregularities in the Dpp gradient seen here were already observed in the negative control (Figure S4); thus, we can assume that the loss of tkv does not have any influence on the Dpp gradient. (PDF) [file pbio.1001111.s006.pdf]

# *tkv*<sup>-</sup>*sax*<sup>-</sup>*brk*<sup>-</sup> LOF clones

## A

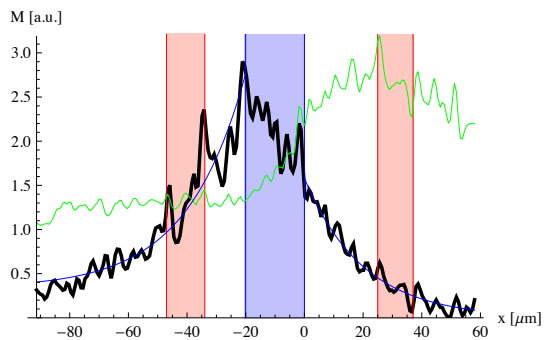

## B

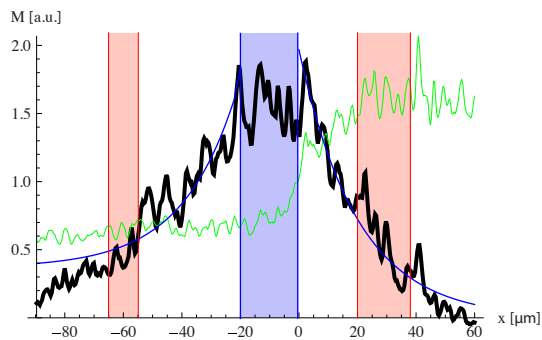

## C

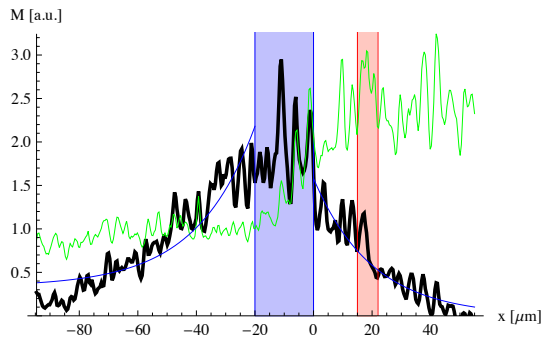

## D

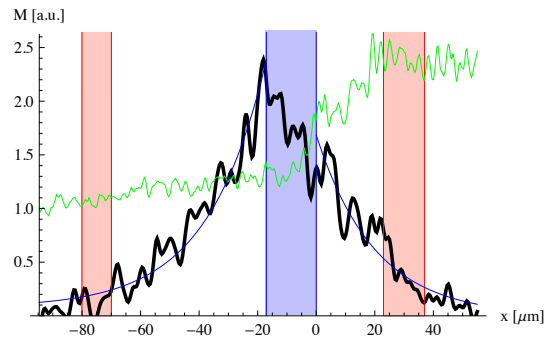

## E

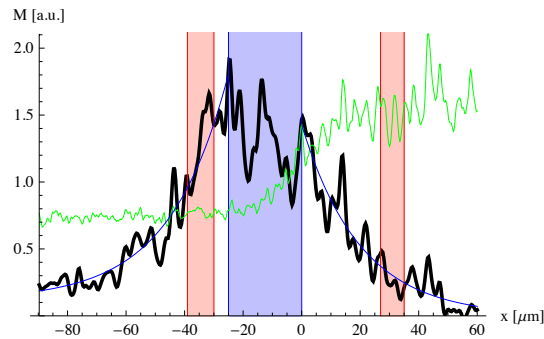

Supplement: Figure S7 — Effect of tkv − brk − clones on the Dpp gradient in sax − wing discs. (A–E) Intensity plots of the Dpp profile from immunofluorescence images of sax mutant 3rd instar wing imaginal discs containing tkv − brk − clones (for an example image, see Figure 4D). The green line represents the GFP:Dpp signal, and the black line represents the intensities of the GFP antibody staining. The Dpp production region is indicated in blue, and the clone region in red. The position x is expressed in µm and the extracted Dpp levels in arbitrary units. sax − tkv − brk − cells do not lead to major alterations of the Dpp gradient. Minor irregularities in the Dpp gradient were already observed in the negative control (Figure S4); thus we can assume that the loss of the two type I receptors sax and tkv does not have any influence on the Dpp gradient. (PDF) [file pbio.1001111.s007.pdf]

# *tkv<sup>-</sup>brk<sup>-</sup>* LOF clones

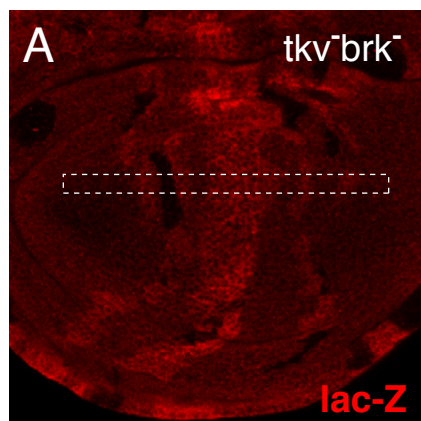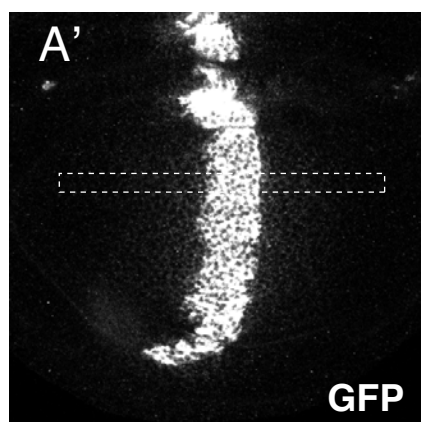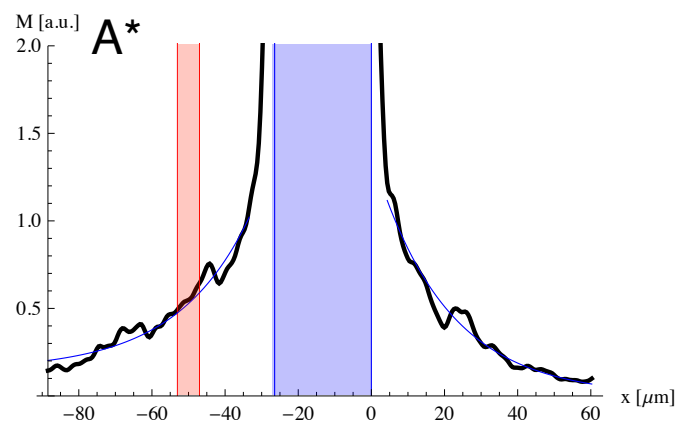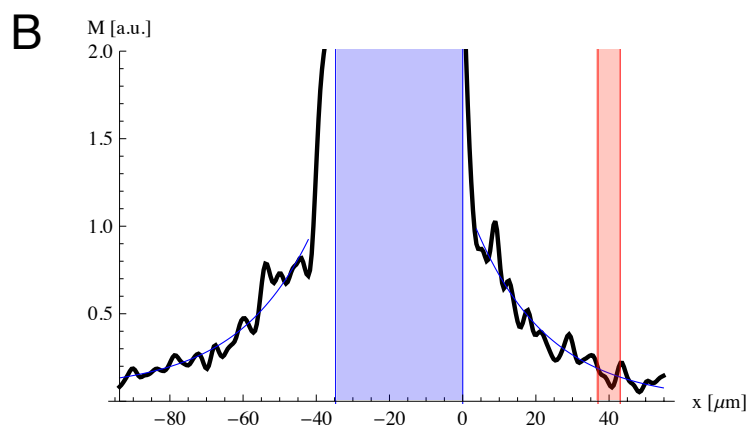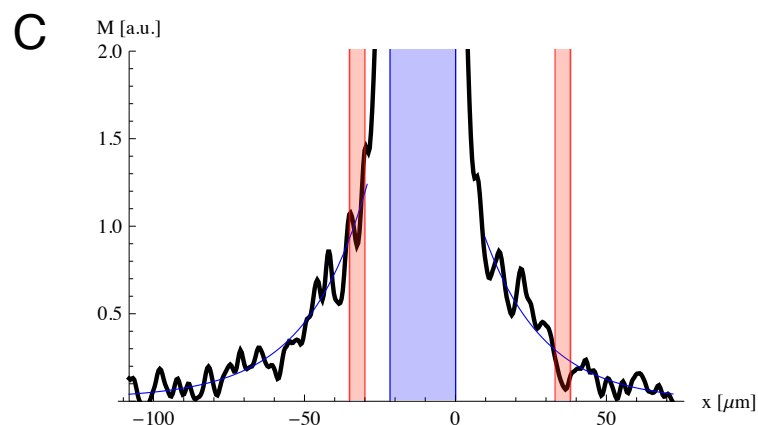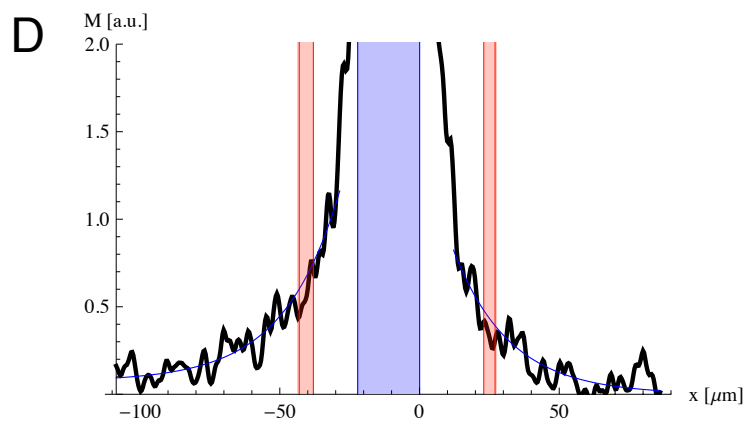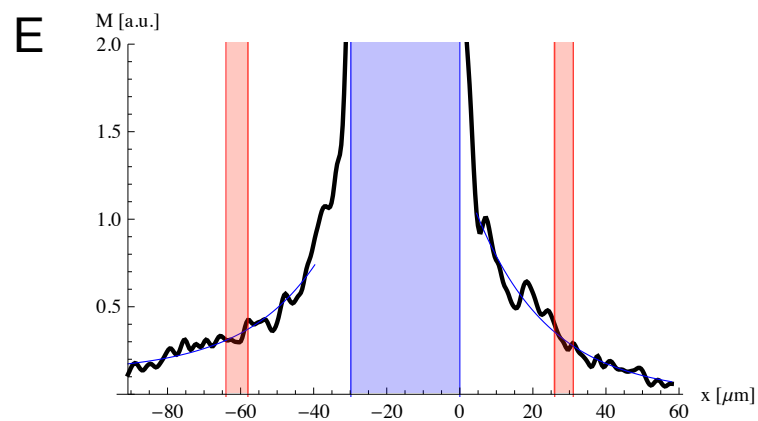

Supplement: Figure S8 — Effect of tkv − brk − clones on the Dpp gradient using a conventional antibody staining protocol. Throughout the article we always analyzed the Dpp gradient using a special antibody staining protocol in order to preserve the extracellular GFP:Dpp pool (see main text and Materials and Methods). In this figure, we examined the effect of tkv − brk − clones on the Dpp gradient using a conventional antibody staining protocol. Confirming the results shown in Figures 4C and S6, also when using the conventional protocol, tkv − brk − clones did not alter the GFP:Dpp gradient. (A, A′) A 3rd instar wing imaginal disc containing tkv − brk − clones. Receptor mutant clones are shown by the loss of lac-Z staining (A), and the GFP:Dpp gradient is visualized using a conventional antibody staining protocol (A′). (A*) GFP:Dpp intensity plot of the marked region of the immunofluorescence image. (B–E) More intensity plots of Dpp profiles of discs containing tkv − brk − clones using conventional antibody staining. (PDF) [file pbio.1001111.s008.pdf]

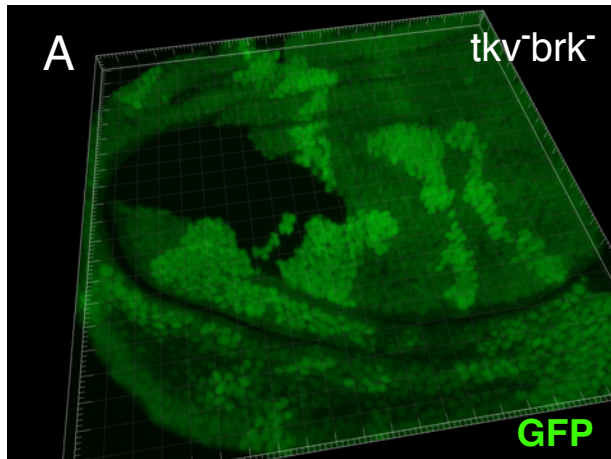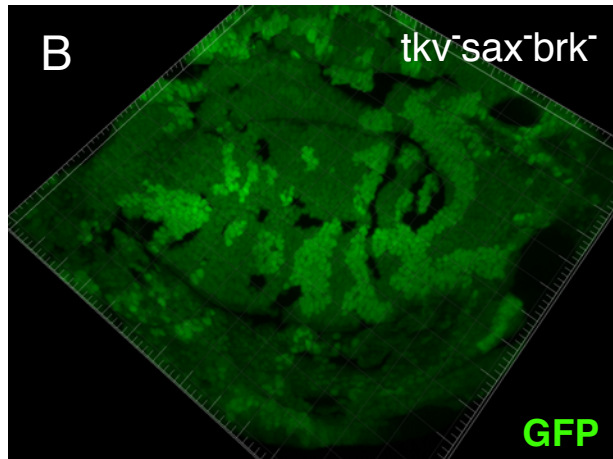

Supplement: Figure S9 — 3-D reconstruction of wing imaginal discs containing clone “islands.” In Figure 5C and 5F, we show patches of wild-type cells fully encircled by tkv − brk − or tkv − sax − brk − mutant cells, which still exhibit substantial Dpp signaling activity (clone “islands”). The rotation of a 3-D reconstruction of the entire z-stack of these discs-images unambiguously shows that the wild-type clone “islands” are fully surrounded by mutant tissue in every z-plane. Dpp therefore has to pass through mutant tissue in order to reach the wild-type patches of cells. Here we show the 3-D reconstructions of the entire z-stack of the wing discs shown in Figure 5C (A) and Figure 5F (B) from one representative angle. (PDF) [file pbio.1001111.s009.pdf]
