# Supplementary material for: Formation of the Long Range Dpp Morphogen Gradient
Source: PLoS Biol. 2011 Jul 26;9(7):e1001111. doi: 10.1371/journal.pbio.1001111 (PMC3144185; doi:10.1371/journal.pbio.1001111)
Supplement: Text S1 — Mathematical and theoretical details of the modeling presented in this article. (PDF) [file pbio.1001111.s010.pdf]

Supporting Text S1:

Analytical modeling of the Dpp profile in wild-type imaginal discs and in discs containing clones mutant for the Dpp receptor

Sascha Dalessi[1,2], Gerald Schwank[3], Aitana Morton de Lachapelle[1,2], Schu-Fee Yang[3], Royhei Yagi[3], Markus Affolter[4], Konrad Basler[3], and Sven Bergmann[1,2]

[1]Department of Medical Genetics, University of Lausanne, Lausanne, Switzerland
[2]Swiss Institute of Bioinformatics
[3]Institute of Molecular Life Sciences, University of Zurich, Zurich, Switzerland
[4]Biozentrum der Universität Basel, Basel, Switzerland

Note: The order of the authors has been modified in this Theoretical Supporting Information to take into account the different contributions to the theoretical modeling

Contents

1 Introduction **3**

2 Dpp wild-type profile **3**
2.1 General wild-type model 3
2.2 Linearization of the differential equations 4
2.3 Analytical expressions for the finite size Dpp production region 6

3 Limit scenarios and parametrical study **7**
3.1 Introduction 7
3.2 Total Dpp mainly external 8
3.3 Total Dpp mainly bound 8
3.4 Total Dpp mainly internalized 9

4 Modeling clones **9**
4.1 *tkv* clones model 9
4.2 External component 11
4.3 Bound component 12
4.4 Internalized component 12

5 Analysis of the clonal effects **13**
5.1 Introduction 13
5.2 Total Dpp mainly external 14
5.3 Total Dpp mainly bound 14
5.4 Total Dpp mainly internalized 15

6 Qualitative comparison with LOF experiments **18**

7 A simplified external-bound model **19**
7.1 Introduction 19
7.2 Total Dpp profile 19

8 Quantitative comparison with GOF experiments **21**

9 Study of the non-linear contributions **23**
9.1 Numerical resolution 24
9.2 Power expansion solution 26

10 Conclusion **27**

1 Introduction

In this supplementary information we present all the mathematical and theoretical details of the modeling presented in the main paper (see Box 1). The technique used for solving the differential equations was developed and reported in a previous work (Dalessi et al., in preparation).
In Section 2, we discuss in more detail the general model and the corresponding set of 1D differential equations describing the Dpp steady state gradient in wild-type (wt) tissues (Dpp external, Tkv-bound and internalized components) as well as the approximation allowing to simplify the problem (linearization) and to obtain explicit analytical solutions. In a first step (cf. Section 3), we investigate three limit case scenarios and look for the corresponding sets of parameters. In these scenarios we assume that the total Dpp is mainly external, mainly Tkv-bound or mainly internalized.
In the *tkv* clonal regions, the number of receptors is altered and the three Dpp components are affected via a modification of the effective binding rate and transcytosis. In Section 4 we present the analytical expressions for the profiles in the presence of *tkv* clones. In Sections 5 and 6, we show (graphically) the effect of the clones within the three limit scenarios and compare them qualitatively to the loss-of-function (LOF) experimental data.
In Section 7 we present a simplified model which completely neglects transcytosis and, in Section 8, we exploit quantitative data extracted from the gain-of-function (GOF) experiments to further refine our initial parameter choice. Finally, in Section 9, we compare the numerical solution of the initial non-linear problem to our simplified analytical solution.

2 Dpp wild-type profile

2.1 General wild-type model

The total Dpp profile $M_{tot}(x)$ has three distinguished components: external Dpp, Tkv-bound Dpp and internalized Dpp.
The external Dpp $M_e(x)$ diffuses in the extracellular medium and can bind to the Tkv receptors. The bound Dpp $M_b(x)$ can unbind or be internalized. The internalized Dpp $M_i(x)$ is degraded or transported to the neighboring cell by transcytosis. The set of differential equations governing the three components at steady state, already presented in the main text (cf. Box 1),

reads

$$\tfrac{d}{dt}M_e(x) = 0 = D_e M_e''(x) - k_+ T(x) M_e(x) + k_- M_b(x) + S(x) \quad (1)$$
$$\tfrac{d}{dt}M_b(x) = 0 = k_+ T(x) M_e(x) - k_- M_b(x) - \kappa M_b(x) \quad (2)$$
$$\tfrac{d}{dt}M_i(x) = 0 = D_i M_i''(x) - \alpha_i M_i(x) + \kappa M_b(x). \quad (3)$$

$T(x) = T_0 - M_b(x)$ represents the local number of free receptors (with T_0 being the homogeneous local total number of receptors), D_e the extracellular diffusion constant, k_+ and k_- the binding/unbinding rates, $S(x)$ refers to the morphogen production region, κ is the internalization rate and α_i represents the internal linear degradation. Finally, we also assume that transcytosis can be described in a diffusive way by introducing an "effective internal diffusion constant" D_i (see Bollenbach et al. [1]). The total Dpp profile is given by $M_{tot}(x) = M_e(x) + M_b(x) + M_i(x)$.

Assuming $T(x) > 0$, the first differential equation can be uncoupled using Eq. (2) and the other two equations solved step-by-step:

$$-D_e M_e''(x) + \frac{\kappa k_+ T_0 M_e(x)}{k_+ M_e(x) + (k_- + \kappa)} = S(x) \quad (4)$$
$$M_b(x) = \frac{k_+ T_0 M_e(x)}{k_+ M_e(x) + (k_- + \kappa)} \quad (5)$$
$$-D_i M_i''(x) + \alpha_i M_i(x) = \kappa M_b(x). \quad (6)$$

This problem, however, requires a numerical solving because the term $T(x)M_e(x)$ induces a non-linearity in the equations.

2.2 Linearization of the differential equations

Under the assumption of a large number of receptors ($T_0 >> \max(M_b(x))$), the number of free receptors is almost constant. Setting $T(x) \equiv T_0$, Eqs. (4)-(6) become linear and reduce to

$$-D_e M_e''(x) + \alpha_e M_e(x) = S(x) \quad (7)$$
$$M_b(x) = \frac{\alpha_e}{\kappa} M_e(x) \quad (8)$$
$$-D_i M_i''(x) + \alpha_i M_i(x) = \kappa M_b(x), \quad (9)$$

where we introduced an "external effective degradation constant"

$$\alpha_e \doteqdot \frac{\kappa k_+ T_0}{k_- + \kappa}. \quad (10)$$

We note that Eq. (7) has the same form as a standard linear 1D Ordinary Differential Equation (ODE) describing the steady state profile of a diffusive process (diffusion constant D_e) with the linear degradation rate α_e. In our model, however, the effective degradation term $\alpha_e M_e(x)$ corresponds to the loss of the external morphogen component through binding to the Tkv receptors and not to a real degradation term. The new parameter α_e can therefore be understood as an "effective binding rate". Furthermore, Eq. (8) shows that the bound Dpp is proportional to the external Dpp and represents a balance between the effective degradation and the internalization $\kappa M_b(x)$. Finally, the last equation (9) is again a linear diffusion-degradation differential equation with degradation rate α_i. Its source term, namely the internalization term $\kappa M_b(x)$, is not anymore localized inside the production region but is completely delocalized over the full wing imaginal disc pouch. This kind of differential equations can nevertheless be solved analytically and all the mathematical tools are reported in our previous work (Dalessi et al., in preparation). In the particular case of no transcytosis at all (i.e. $D_i = 0$), the internalized Dpp also becomes linearly dependent on the external Dpp: $M_i(x) = \frac{\alpha_e}{\alpha_i} M_e(x)$ and the total Dpp concentration $M_{tot}(x)$ can be described by a unique ODE, namely

$$-D_e M''_{tot}(x) + \alpha_e M_{tot}(x) = \alpha_e \left(\frac{1}{\alpha_e} + \frac{1}{\kappa} + \frac{1}{\alpha_i} \right) S(x).$$

Introducing, for $\beta = e$ and i, the Green functions

$$G_\beta(x) = \frac{e^{-|x|/\lambda_\beta}}{2\alpha_\beta \lambda_\beta},$$

where $\lambda_\beta = \sqrt{D_\beta/\alpha_\beta}$ are the decay lengths of the exponentials, we obtain the following integral expressions for the Dpp components (for more details we refer to Dalessi et al., in preparation):

$$M_e(x) = \int_{\mathbb{R}} dy\, G_e(x-y) S(y) \tag{11}$$

$$M_b(x) = \frac{\alpha_e}{\kappa} M_e(x) \tag{12}$$

$$M_i(x) = \kappa \int_{\mathbb{R}} dy\, G_i(x-y) M_b(y) = \alpha_e \int_{\mathbb{R}} dy\, G_{ie}(x-y) S(y) \tag{13}$$

where $G_{ie}(x-y) \doteqdot \int_{\mathbb{R}} dz\, G_i(x-z) G_e(z-y) = \frac{\alpha_e \lambda_e^2 G_e(x-y) - \alpha_i \lambda_i^2 G_i(x-y)}{\alpha_e \alpha_i (\lambda_e^2 - \lambda_i^2)}$.
The effect of an increase of the homogeneous local total number of receptors with the distance from the production region has been studied numerically. As it only affects in a minor way the Dpp profiles, in this simplified model we therefore always assume a constant value T_0.

2.3 Analytical expressions for the finite size Dpp production region

In the literature, morphogen sources (production regions) are often modeled as a point source at the origin $S^{\delta}(x) = s_0\delta(x)$, with s_0 being the molecular production rate per time unit. In such a case, the external Dpp at steady state is a pure decreasing exponential $M_e^{\delta}(x) = s_0 G_e(x)$ and the internalized Dpp expression reduces to $M_i^{\delta}(x) = s_0 G_{ie}(x)$. Despite its analytical simplicity, this choice is however not very adequate, because the first derivative of $M_e^{\delta}(x)$ presents a discontinuity at the source where we expect a smoother behavior. To account for this, we consider a constant production of Dpp in an extended region $[-x_0, 0]$ corresponding to a source

$$S^{\theta}(x) = \tfrac{s_0}{x_0}\theta(-x)\theta(x + x_0).$$

The analytical solutions for the external and internalized Dpp are obtained from

$$M_{\beta}^{\theta}(x) = \frac{1}{x_0}\int_{-x_0}^{0} dy\, M_{\beta}^{\delta}(x - y)$$

and can be expressed in a condensed way as follows:

$$\begin{aligned} M_e^{\theta}(x < -x_0) &= -f_e^{+}(x) \\ M_e^{\theta}(x \in [-x_0, 0]) &= g_e(x) \\ M_e^{\theta}(x > 0) &= f_e^{-}(x) \end{aligned} \tag{14}$$

$$\begin{aligned} M_i^{\theta}(x < -x_0) &= -a\left(\lambda_e^2 f_e^{+}(x) - \lambda_i^2 f_i^{+}(x)\right) \\ M_i^{\theta}(x \in [-x_0, 0]) &= a\left(\lambda_e^2 g_e(x) - \lambda_i^2 g_i(x)\right) \\ M_i^{\theta}(x > 0) &= a\left(\lambda_e^2 f_e^{-}(x) - \lambda_i^2 f_i^{-}(x)\right), \end{aligned} \tag{15}$$

where we defined

$$\begin{aligned} a &= \frac{\alpha_e}{\alpha_i(\lambda_e^2 - \lambda_i^2)} \\ f_{\beta}^{\kappa}(x) &= \frac{s_0}{2x_0\alpha_e}\left(1 - e^{\kappa x_0/\lambda_\beta}\right) e^{\kappa x/\lambda_\beta} \\ g_{\beta}(x) &= \frac{s_0}{2x_0\alpha_e}\left(2 - e^{x/\lambda_\beta} - e^{-(x+x_0)/\lambda_\beta}\right). \end{aligned}$$

We note in (14) that outside the production region, the profile $M_e^{\theta}(x)$ is purely exponential with the same decay length λ_e as in $M_e^{\delta}(x)$ (Dalessi et al., in preparation). The only qualitative difference between the two profiles is

the flattening inside the production region (vanishing derivative at the center of the source $x = -x_0/2$).
An alternative way to model an extended source would be to consider a normal (Gaussian) distribution: in this case, the production region has no clear-cut boundaries and the profile is no longer a pure exponential. Nevertheless, far enough from the source, the corresponding external Dpp profile is very similar to an exponential (Dalessi et al., in preparation). In the following sections we always model a finite size production region and we therefore omit the redundant θ labeling.

3 Limit scenarios and parametrical study

3.1 Introduction

Our model involves six parameters that are not directly constrained experimentally: α_β, λ_β (for $\beta = e, i$), κ and x_0. Recalling the relationship $\lambda_\beta = \sqrt{D_\beta/\alpha_\beta}$, we can either use the decay lengths λ_β or the diffusion constants D_β as free parameters.
Since it is very hard to determine experimentally the value of these parameters, we consider some constraints to reduce the parameter space. We first notice that the total relative abundances of each component $I_\beta = \int_{\mathbb{R}} dx\, M_\beta(x)$, for $\beta = e$, b and i, are given by

$$I_e = \frac{s_0}{\alpha_e}, \qquad I_b = \frac{s_0}{\kappa}, \qquad I_i = \frac{s_0}{\alpha_i}.$$

Thereby, considering a global normalization $I_{tot} = I_e + I_b + I_i = 1$, the three constants α_e, κ and α_i are univocally determined imposing the relative abundance of each component. For the numerical applications, we set the width of the production region to $x_0 = 0.15L$ and fix the last two parameters, λ_e and λ_i, imposing that (i) the total Dpp profile $M_{tot}(x)$ outside the source is an exponentially decaying profile with decay length $\lambda = 0.2L$, where L is the half-length of the *Drosophila* wing disc pouch (see Kicheva et al. [2]), and that (ii) every component displays a strong confinement inside the pouch $r_\beta = M_\beta(-L)/M_\beta(-x_0/2) < 2\%$.

We first investigate three limit cases: the total Dpp is (i) mainly external, (ii) mainly bound to the Tkv receptors or (iii) mainly internalized. In the next section (see Section 4), we obtain analytical expressions for the Dpp profiles in a tissue with *tkv* clones. The three limit cases give rise to three distinct scenarios which are compared to the experimental data.

3.2 Total Dpp mainly external

In this first case, we set $I_e = 0.8$, $I_b = I_i = 0.1$ leading to $\alpha_e = \frac{5}{4}s_0$, $\kappa = \alpha_i = 10s_0$. We note that the external Dpp production rate s_0 cancels out in Eqs. (14) and (15). Since the total Dpp profile is dominated by its external component which decreases exponentially outside the production region, assuming $\lambda_e \cong \lambda = 0.2L$ is a good approximation. Finally, the maximal allowed transcytosis rate to ensure a good confinement $r_i < 2\%$ of $M_i(x)$ is given by $\lambda_i^{Max} \cong \frac{1}{2}\lambda_e$. The resulting Dpp profiles are presented in Fig. 1. We point out that in this limit scenario, we allow transcytosis to range between $D_i = 0$ (no transcytosis) to $D_i^{Max} = 0.1L^2 s_0$ (maximal transcytosis). However, comparing Figs. 1 **(a)** and **(b)**, the total Dpp profile is almost not affected because the value of λ_i only influences the internalized component which represents a minor fraction of the total Dpp. In **(a)**, we also note that the profiles $M_b(x)$ and $M_i(x)$ are identical. This is because, in absence of transcytosis, $M_i(x) = \frac{\kappa}{\alpha_i}M_b(x) = M_b(x)$. By contrast, in **(b)**, the profile $M_i(x)$ (blue line) is flattened due to the transcytosis (internal diffusion).

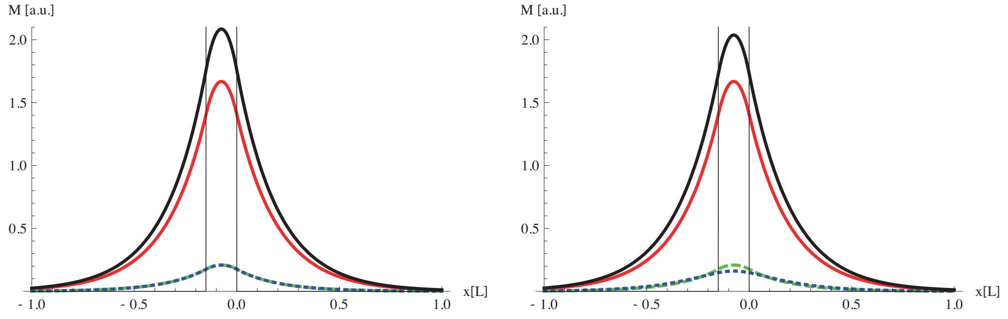

(a) **(b)**

Figure 1: Dpp profile (solid black line) and its external (solid red line), bound (dashed green line) and internalized (dotted blue line) components for the limit scenario dominated by the external component. The source width has been set to $x_0 = 0.15L$ (vertical thin lines). The parameters are $\lambda_e = 0.2L$, $\alpha_e = \frac{5}{4}s_0$, $\kappa = \alpha_i = 10s_0$. We consider no transcytosis ($\lambda_i = 0$) in **(a)** and maximal transcytosis $\lambda_i^{Max} = \frac{1}{2}\lambda_e$ in **(b)**.

3.3 Total Dpp mainly bound

Here $I_e = I_i = 0.1$, $I_b = 0.8$ yielding $\kappa = \frac{5}{4}s_0$, $\alpha_e = \alpha_i = 10s_0$, $\lambda_e = 0.2L$, $\lambda_i^{Max} \cong \frac{1}{2}\lambda_e$. The profiles correspond to Fig. 1 but with red and green lines exchanged.

3.4 Total Dpp mainly internalized

We have $I_e = I_b = 0.1$, $I_i = 0.8$ leading to $\alpha_i = \frac{5}{4}s_0$, $\kappa = \alpha_e = 10s_0$. In this case, the approximation $\lambda_e \cong \lambda = 0.2L$ is not valid anymore and a numerical exponential fitting procedure of $M_{tot}(x > 0)$ is required. This leads to a set of allowed pairs (λ_e, λ_i) as shown in Fig. 2. In Fig. 3, we present the resulting

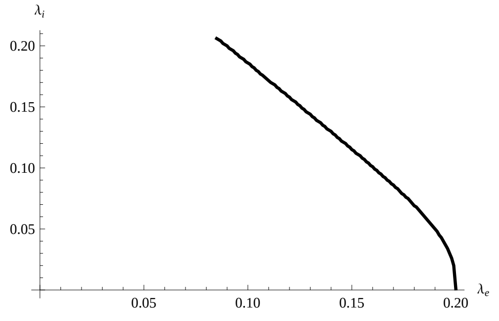

Figure 2: Relationship between λ_e and λ_i under the requirement of a good numerical exponential fit with decay length $\lambda = 0.2L$ of $M_{tot}(x)$ outside the production region when the total Dpp is mainly internalized.

profiles with no transcytosis **(a)** and maximal transcytosis **(b)**. Transcytosis strongly affects, directly or indirectly, each Dpp component because the global diffusion is shared between pure external diffusion (characterized by the parameter λ_e) and transcytosis (parameter λ_i). Thereby, strong transcytosis imposes a smaller external diffusion constant. The total Dpp profile is however almost identical in all cases because we imposed an exponential decay with $\lambda = 0.2L$. As $\alpha_e = \kappa$, the external and Tkv-bound Dpp components are always identical (cf. Eq. (12)).

4 Modeling clones

4.1 *tkv* clones model

A clonal experiment involves a mutation in a subset of clustered cells in tissue. This can be modeled mathematically by considering that one or more parameters (like the diffusion or degradation constant) are no longer uniform in space but altered in the clonal region. Although in a general case this is a non-trivial problem to solve (non-constant coefficient differential equations), a 1D clone model at steady state can nevertheless be studied analytically by considering different constant values of the parameters outside and inside the

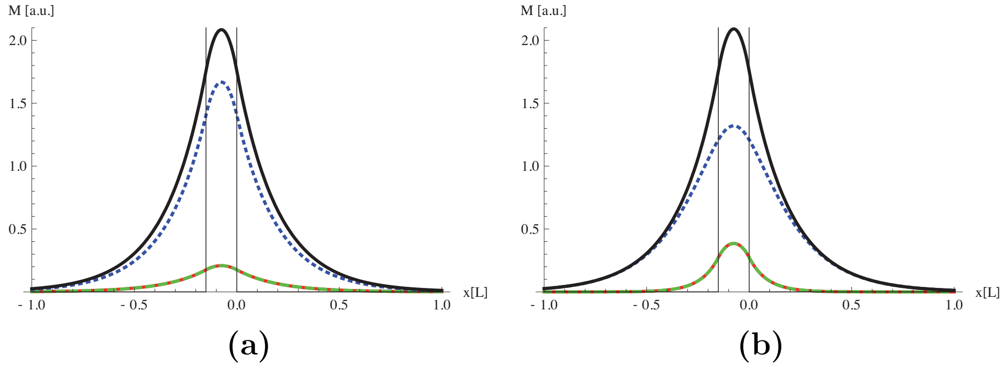

Figure 3: Dpp profile (solid black line) and its external (solid red line), bound (dashed green line) and internalized (dotted blue line) components for the limit scenario dominated by the internalized component. The source width has been set to $x_0 = 0.15L$ (vertical thin lines). The parameters are $\alpha_i = \frac{5}{4}s_0$, $\kappa = \alpha_e = 10s_0$. We consider no transcytosis ($\lambda_i = 0$, $\lambda_e = 0.2$) in **(a)** and maximal transcytosis ($\lambda_i \cong 0.2$, $\lambda_e \cong 0.09$) in **(b)**.

clone $\mathcal{C} = [x_1, x_2]$ and solving separately the differential equations in front, inside and behind the clone. Flux conservation and function continuity at the boundaries allow to connect the local solutions and fix the constants related to the homogeneous solutions of the differential equations.

In this study, we performed experiments with *tkv* clones affecting the number of receptors ($T_0^{clone} = nT_0$). In the LOF experiments, we assume that inside the clone we have no receptors at all ($n = 0$) whereas in the GOF experiments, the number of receptors increases ($n > 1$). Since $\alpha_e \sim T_0$ (see Eq. (10)), *tkv* clones affect the effective degradation constant leading to $\alpha_e^{clone} = n\alpha_e$. Furthermore, since we consider that transcytosis is receptor-mediated, the effective internal diffusion constant D_i is also affected by the clone. Assuming a linear dependence $D_i \sim T_0$, this leads to $D_i^{clone} = nD_i$ inside the clone.
In [3], Eldar and Barkai presented an extensive study of clone effects on morphogen profiles. In this work, we focus on a 1D model at steady state taking into account the effects of receptor-mediated transcytosis.

From the set of differential equations (7)-(9), we see that the profiles affected by the clone $\bar{M}_\beta(x)$, involving the space-dependent parameters $\alpha_e(x)$

and $D_i(x)$, are solution of

$$-D_e\bar{M}_e''(x) + \alpha_e(x)\bar{M}_e(x) = S(x) \quad (16)$$

$$\bar{M}_b(x) = \frac{\alpha_e(x)}{\kappa}\bar{M}_e(x) \quad (17)$$

$$-\left(D_i(x)\bar{M}_i'(x)\right)' + \alpha_i\bar{M}_i(x) = \kappa\bar{M}_b(x). \quad (18)$$

4.2 External component

In this case (see Eq. (16)), the effective degradation is affected since inside the clone $\mathcal{C} = [x_1,\, x_2]$ we have $\alpha_e^{clone} = n\alpha_e \;\Rightarrow\; \lambda_e^{clone} = \frac{1}{\sqrt{n}}\lambda_e$.
Focusing on the particular case of a clone with $0 < x_1 < x_2$, the general solution for the profile $\bar{M}_e(x)$ reads

$$\begin{aligned}
\bar{M}_e(x < x_1) &= M_e(x) + c_1 e^{-x/\lambda_e} + c_2 e^{x/\lambda_e} \\
\bar{M}_e(x \in \mathcal{C}) &= c_3 e^{-\sqrt{n}x/\lambda_e} + c_4 e^{\sqrt{n}x/\lambda_e} \\
\bar{M}_e(x > x_2) &= c_5 e^{-x/\lambda_e} + c_6 e^{x/\lambda_e}.
\end{aligned} \quad (19)$$

The particular solution $M_e(x)$ corresponds to the wt profile defined in Eq. (14). The constants c_1 and c_6 are set to zero to satisfy the conditions $\bar{M}_e(\pm\infty) = 0$. The four remaining n-dependent constants c_2 to c_5 are fixed by imposing functional continuity and flux conservation conditions at the two clone boundaries x_j, $j = 1, 2$. For the external component, flux conservation reduces to continuity of the first derivative because the diffusion constant is not affected by the clone and $J_e(x) = -D_e(x)\bar{M}_e'(x) = -D_e\bar{M}_e'(x)$.
Introducing

$$\begin{aligned}
f_0 &= f_e^-(x_1)\left(e^{2\sqrt{n}x_2/\lambda_e}\left(\sqrt{n}+1\right)^2 - e^{2\sqrt{n}x_1/\lambda_e}\left(\sqrt{n}-1\right)^2\right)^{-1} \\
f(a_1, a_2) &= 2f_0 e^{(a_1x_1 - a_2x_2)/\lambda_e},
\end{aligned}$$

the coefficients read

$$\begin{aligned}
c_2 &= (n-1)f_0 e^{-x_1/\lambda_e}\left(e^{2\sqrt{n}x_1/\lambda_e} - e^{2\sqrt{n}x_2/\lambda_e}\right) \\
c_3 &= (\sqrt{n}+1)f(\sqrt{n}, -2\sqrt{n}) \\
c_4 &= (\sqrt{n}-1)f(\sqrt{n}, 0) \\
c_5 &= 2\sqrt{n}f(\sqrt{n}, -1-\sqrt{n}).
\end{aligned}$$

As expected, for $n = 1$ the effects of the clone vanish and $\bar{M}_e(x)$ reduces to the wt profile given in (14).

The particular case $n = 0$, related to the LOF experiments, can be obtained taking the limits

$$\lim_{n\to 0} c_2 = g_0 e^{-x_1/\lambda_e}(x_1 - x_2)$$
$$\lim_{n\to 0} \left(c_3 e^{-x/\lambda_e} + c_4 e^{x/\lambda_e}\right) = 2g_0\left(x - (x_2 + \lambda_e)\right)$$
$$\lim_{n\to 0} c_5 = -2\lambda_e g_0 e^{x_2/\lambda_e},$$

where $g_0 = f_e^-(x_1)/(x_1 - x_2 - 2\lambda_e)$. As expected, we find that the solution inside the clone becomes linear since the differential equation (16) reduces to $D_e \bar{M}_e''(x \in \mathcal{C}) = 0$.

The analytical solution $\bar{M}_e^l(x)$ for a clone located on the left part of the source ($\mathcal{C}^l = [-x_0 - x_2, -x_0 - x_1]$) can be deduced directly from the previous case, $\bar{M}_e^l(x) = \bar{M}_e(-x_0 - x)$, because the wt problem is symmetric with respect to the center of the source $x = -x_0/2$. A clone inside or crossing the production region requires a modification of the set of equations (19) and a consequent new analytical resolution because additional non-vanishing particular solutions appear. Multiple clones can also be modeled solving the differential equation separately in front, inside and behind each clone.

4.3 Bound component

From Eq. (17), we directly obtain $\bar{M}_b(x)$ from $\bar{M}_e(x)$:

$$\bar{M}_b(x \notin \mathcal{C}) = \frac{\alpha_e}{\kappa}\bar{M}_e(x)$$
$$\bar{M}_b(x \in \mathcal{C}) = n\frac{\alpha_e}{\kappa}\bar{M}_e(x).$$

For $n = 0$, the profile levels drop to zero inside the clone, $\bar{M}_b(x \in \mathcal{C}) = 0$.

4.4 Internalized component

The procedure is similar to that presented in Section 4.2, but assuming $D_i^{clone} = nD_i \Rightarrow \lambda_i^{clone} = \sqrt{n}\lambda_i$:

$$\bar{M}_i(x < x_1) = \bar{m}_i(x) + d_2 e^{x/\lambda_i}$$
$$\bar{M}_i(x \in \mathcal{C}) = \bar{m}_i^{\mathcal{C}}(x) + d_3 e^{-x/\sqrt{n}\lambda_i} + d_4 e^{x/\sqrt{n}\lambda_i}$$
$$\bar{M}_i(x > x_2) = \bar{m}_i(x) + d_5 e^{-x/\lambda_i},$$

where

$$\begin{aligned}\bar{m}_i(x) &= \kappa \int_{\mathbb{R}} dy\, G_i(x-y)\bar{M}_b(y) \qquad \text{and} \\ \bar{m}_i^{\mathcal{C}}(x) &= \kappa \int_{\mathbb{R}} dy\, G_i^{\mathcal{C}}(x-y)\bar{M}_b(y)\end{aligned} \tag{20}$$

are the particular solutions outside and inside the clone, with

$$G_i^{\mathcal{C}}(x) = \frac{e^{-|x|/\sqrt{n}\lambda_i}}{2\sqrt{n}\alpha_i\lambda_i}.$$

The coefficients read

$$\begin{aligned}
d_2 &= h_0 e^{-\frac{x_1}{\lambda_i}} \left(2\sqrt{n} e^{\frac{x_1+x_2}{\sqrt{n}\lambda_i}} \Delta^1_{2,1} + (\sqrt{n}-1) e^{\frac{2x_1}{\sqrt{n}\lambda_i}} \Delta^{-1}_{1,n} + (\sqrt{n}+1) e^{\frac{2x_2}{\sqrt{n}\lambda_i}} \Delta^1_{1,n} \right) \\
d_3 &= h_0 e^{\frac{x_1+x_2}{\sqrt{n}\lambda_i}} \left((\sqrt{n}-1) e^{\frac{x_1}{\sqrt{n}\lambda_i}} \Delta^1_{2,1} + (\sqrt{n}+1) e^{\frac{x_2}{\sqrt{n}\lambda_i}} \Delta^{-1}_{1,1} \right) \\
d_4 &= h_0 \left((\sqrt{n}-1) e^{\frac{x_1}{\sqrt{n}\lambda_i}} \Delta^{-1}_{1,1} + (\sqrt{n}+1) e^{\frac{x_2}{\sqrt{n}\lambda_i}} \Delta^1_{2,1} \right) \\
d_5 &= h_0 e^{\frac{x_2}{\lambda_i}} \left(2\sqrt{n} e^{\frac{x_1+x_2}{\sqrt{n}\lambda_i}} \Delta^{-1}_{1,1} - (\sqrt{n}-1) e^{\frac{2x_1}{\sqrt{n}\lambda_i}} \Delta^1_{2,n} + (\sqrt{n}+1) e^{\frac{2x_2}{\sqrt{n}\lambda_i}} \Delta^{-1}_{2,n} \right),
\end{aligned}$$

with

$$\begin{aligned}
h_0 &= \left(e^{\frac{2x_1}{\sqrt{n}\lambda_i}} (\sqrt{n}-1)^2 - e^{\frac{2x_2}{\sqrt{n}\lambda_i}} (\sqrt{n}+1)^2 \right)^{-1} \\
\Delta^k_{j,m} &= \sqrt{m}(M_i^{\mathcal{C}}(x_j) - M_i(x_j)) + k\lambda_i(n\tfrac{d}{dx}M_i^{\mathcal{C}}(x_j) - \tfrac{d}{dx}M_i(x_j)).
\end{aligned}$$

In the particular case where $n = 0$, we obtain $\bar{M}_i(x \in \mathcal{C}) \equiv 0$ since inside the clone, both the source term $\kappa\bar{M}_b(x)$ and the diffusion constant are zero. The particular solution reduces to $\bar{m}_i^0(x) \equiv \lim_{n\to 0} \bar{m}_i(x)$ and the constants d_2 and d_5 to

$$\begin{aligned}
\lim_{n\to 0} d_2 &= -\lambda_i e^{-x_1/\lambda_i} \tfrac{d}{dx}\bar{m}_i^0(x_1) \\
\lim_{n\to 0} d_5 &= \lambda_i e^{x_2/\lambda_i} \tfrac{d}{dx}\bar{m}_i^0(x_2).
\end{aligned}$$

ensuring vanishing derivatives at the clone boundaries $\frac{d}{dx}\bar{M}_i(x_1) = \frac{d}{dx}\bar{M}_i(x_2) = 0$.

5 Analysis of the clonal effects

5.1 Introduction

In this section, we present graphically the effect of the clones on the total Dpp profile for the three limit scenarios discussed in Section 3. For the LOF

we present the figures for the particular case $n = 0$ (no receptors inside the clone), and for the GOF we consider $n = 10$.
We recall that our parameters were determined based on the wt profiles, thus the relative ratios of the corresponding components are no longer preserved in the clone profiles. Indeed, clones affect the effective degradation constant α_e leading to a global net increase or decrease of external Dpp. For the Tkv-bound and internalized components, however, the total number of molecules is preserved.

5.2 Total Dpp mainly external

The parameters corresponding to this first limiting case (80% of the total Dpp in the wt profile is external, 10% is Tkv-bound and 10% is internalized) were calculated in section 3.2 and the corresponding wt profiles are shown in Fig. 1. In Fig. 4, we present the modified Dpp profiles in the presence of a LOF and GOF clone respectively.
We first notice in Figs. 4 **(a)** and **(b)**, for the LOF case, that the sets of parameters corresponding to $D_i = 0$ (no transcytosis) and maximal transcytosis give qualitatively very similar results. This is because the internalized component (dotted blue lines), which is affected by transcytosis, only represents 10% of the total Dpp. Furthermore, internalized Dpp levels, as well as Tkv-bound Dpp levels, vanish inside the clone independently of the transcytosis magnitude due to the absence of receptors and transcytosis. The global behavior of the total Dpp profiles show a small decrease inside the clone.
For the GOF case, Figs. 4 **(c)** and **(d)** are slightly different. Instead of a small decrease inside the clone, we obtain a relatively consistent increase of total Dpp levels with the appearance of a peak at the beginning of the clone. In absence of transcytosis, as in the wt profile, the bound and internalized profiles are identical to each other, for both the LOF and GOF cases.

5.3 Total Dpp mainly bound

The parameters corresponding to the second limit case (80% of the total Dpp in the wt profile is Tkv-bound, 10% is external and 10% is internalized), were calculated in section 3.3. Results for the LOF and GOF cases are reported in Fig. 5.
In the LOF case, the total Dpp levels are considerably reduced inside the clone while in the GOF case a very high peak appears at the beginning of the clone. This is a direct consequence of the fact that the bound component, which dominates the global behavior of the profile, is strongly affected by the lack, respectively strong increase, of receptors inside the clone.

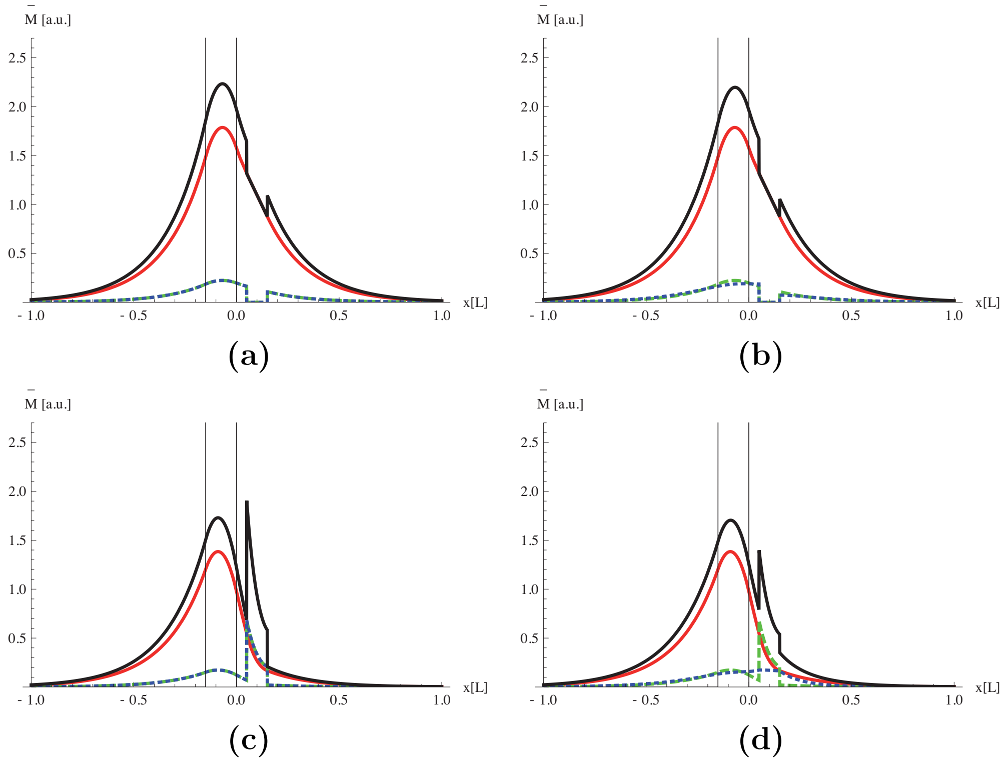

Figure 4: Clone effects on the Dpp profile in the LOF ($n = 0$) **(a)**-**(b)** and GOF ($n = 10$) **(c)**-**(d)** cases. We show the total Dpp (black solid line) and its external (solid red line), bound (dashed green line) and internalized (dotted blue line) components for the scenario dominated by external Dpp. The source width has been set to $x_0 = 0.15L$ (vertical lines) and the clone is located between $x_1 = 0.05L$ and $x_2 = 0.15L$. The parameters are $\lambda_e = 0.2L$, $\alpha_e = \frac{5}{4}s_0$, $\kappa = \alpha_i = 10s_0$. In **(a)** and **(c)** there is no transcytosis and in **(b)** and **(d)** transcytosis is maximal ($\lambda_i^{Max} = \frac{1}{2}\lambda_e$).

5.4 Total Dpp mainly internalized

The parameters corresponding to the last limiting case (80% of the total Dpp in the wt profile is internalized, 10% is external and 10% is Tkv-bound) were calculated in Section 3.4 and the corresponding wt profiles are shown in Fig. 3. Results for the LOF and GOF cases are reported in Fig. 6.

When there is no transcytosis ($\lambda_i = 0$, $\lambda_e = 0.2L$), the behavior of the total Dpp profiles in Fig. 6 **(a)** and **(c)** are identical to those in Fig. 5 **(a)** and **(c)**. In the LOF case with transcytosis, Dpp levels are low after the clone because clone-crossing can only happen externally by pure diffusion ($D_i = 0$).

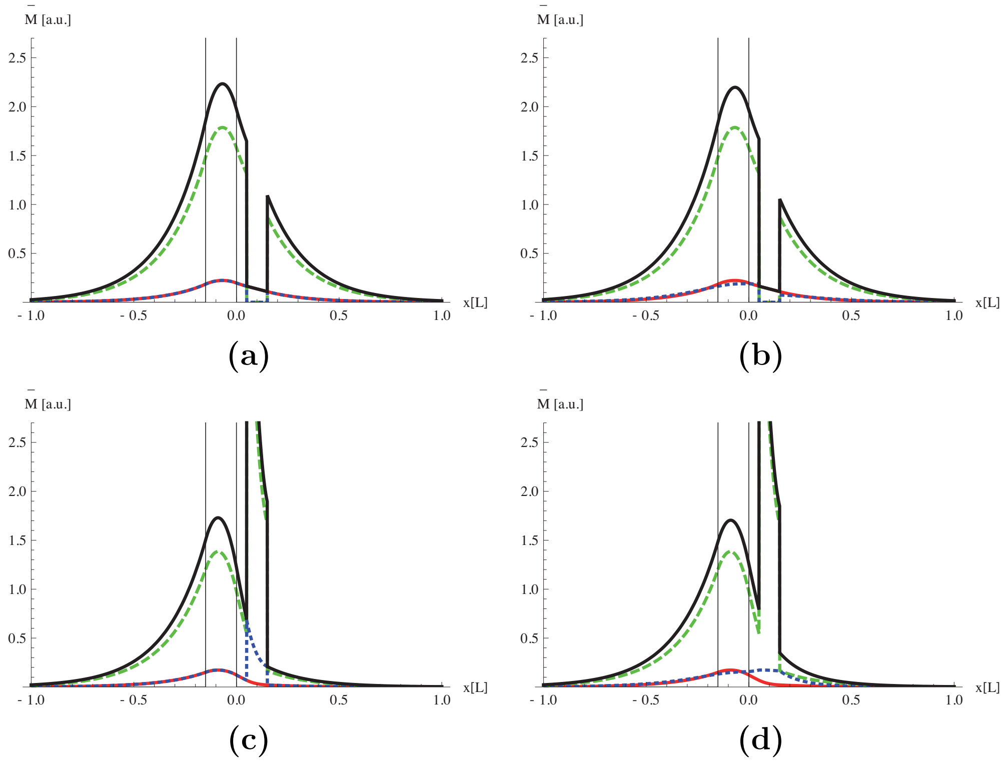

Figure 5: Clone effects on the Dpp profile in the LOF ($n = 0$) **(a)**-**(b)** and GOF ($n = 10$) **(c)**-**(d)** cases. We show the total Dpp (black solid line) and its external (solid red line), bound (dashed green line) and internalized (dotted blue line) components for the scenario dominated by bound Dpp. The source width has been set to $x_0 = 0.15L$ (vertical lines) and the clone is located between $x_1 = 0.05L$ and $x_2 = 0.15L$. The parameters are $\lambda_e = 0.2L$, $\kappa = \frac{5}{4}s_0$, $\alpha_i = \alpha_e = 10s_0$. In **(a)** and **(c)** there is no transcytosis and in **(b)** and **(d)** transcytosis is maximal ($\lambda_i^{Max} = \frac{1}{2}\lambda_e$).

In the GOF case with transcytosis, the effect of strong transcytosis is to attenuate the peak inside the clone and cancel the effect of the clone (internal diffusion from inside to outside the clone). Only for a clone very close to the production region, like in Fig. 6 **(d)**, a small peak is still present.

In our model we always consider that transcytosis is receptor-mediated. A hypothetical theoretical model involving receptor-independent transcytosis would give, as shown in Fig. 7, qualitatively similar results for the GOF case, but a completely different behavior for the LOF case. Indeed, since transcytosis is no longer zero inside the clone, the levels of Dpp inside the

clone can increase via cell-by-cell transport. In a receptor-independent transcytosis model, both LOF and GOF cases would therefore be qualitatively indistinguishable from the scenario where the total Dpp is mainly external (cf. Figs. 4 **(b)** and **(d)**). The analytical expression for the internalized component would reduce to $\bar{M}_i(x) = \bar{m}_i(x)$ (cf. Eq. 20).

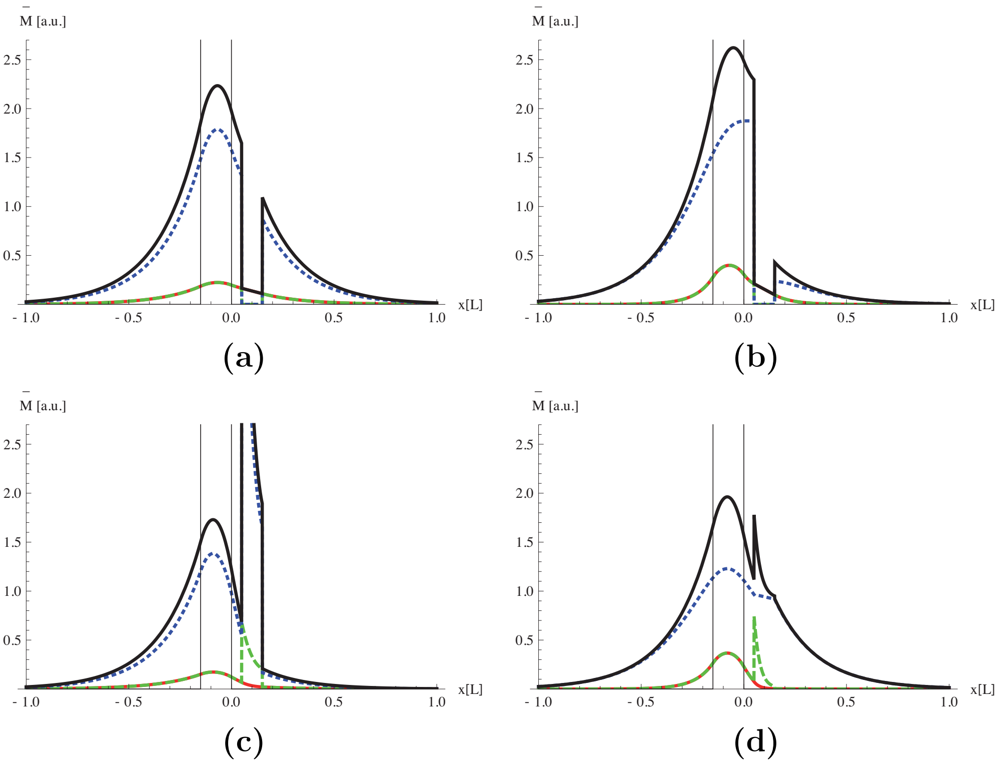

Figure 6: Clone effects on the Dpp profile in the LOF ($n = 0$) **(a)**-**(b)** and GOF ($n = 10$) **(c)**-**(d)** cases. We show the total Dpp (black solid line) and its external (solid red line), bound (dashed green line) and internalized (dotted blue line) components for the scenario dominated by internalized Dpp. The source width has been set to $x_0 = 0.15L$ (vertical lines) and the clone is located between $x_1 = 0.05L$ and $x_2 = 0.15L$. The parameters are $\alpha_i = \frac{5}{4}s_0$, $\kappa = \alpha_e = 10s_0$. In **(a)** and **(c)** there is no transcytosis ($\lambda_i = 0, \lambda_e = 0.2L$) and in **(b)** and **(d)** transcytosis is maximal ($\lambda_i \cong 0.2L, \lambda_e \cong 0.09L$).

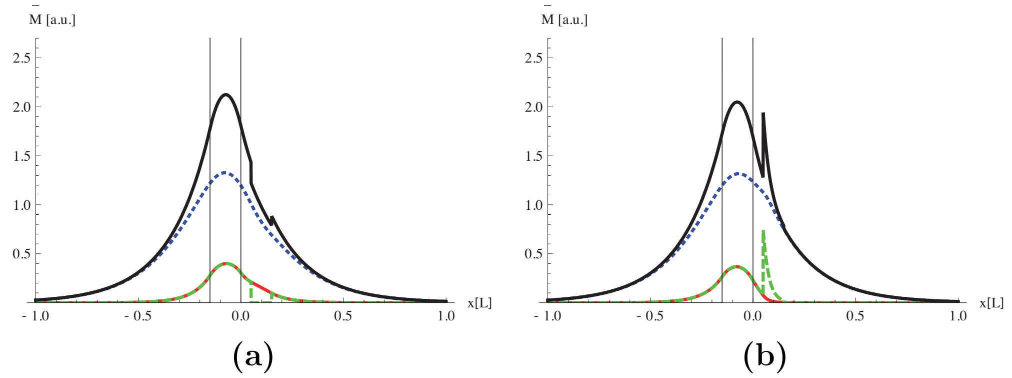

Figure 7: Clone effects on the Dpp profile in the LOF ($n = 0$) **(a)** and GOF ($n = 10$) **(b)** cases for an hypothetical receptor-independent transcytosis model with maximal transcytosis ($\lambda_i \cong 0.2L, \lambda_e \cong 0.09L$). We show the total Dpp (black solid line) and its external (solid red line), bound (dashed green line) and internalized (dotted blue line) components for the scenario dominated by internalized Dpp. The source width has been set to $x_0 = 0.15L$ (vertical lines) and the clone is located between $x_1 = 0.05L$ and $x_2 = 0.15L$. The parameters are $\alpha_i = \frac{5}{4}s_0$, $\kappa = \alpha_e = 10s_0$.

6 Qualitative comparison with LOF experiments

Experimental data for LOF experiments (see Fig. 4 in the main paper and supplementary figures S5 and S6) suggest that there are significant Dpp levels inside and behind the clone. These qualitative evidences allow to reject a model in which the majority of Dpp is internalized and transported via receptor-mediated transcytosis (see Figs. 6 (**b**), low levels inside and behind the clone). We expect therefore a mixing between the other two scenarios, namely the total Dpp being mainly external and mainly bound. Furthermore, non-negligible levels inside the clone suggest that the external component should be the dominating one. As these two scenarios are only marginally affected by transcytosis (see Figs. 4 **(a)**-**(b)** and 5 **(a)**-**(b)**), we conclude that external diffusion, and not receptor-mediated transcytosis, plays the dominant role in Dpp gradient formation.

In the following sections, we therefore consider a simplified model (see Section 7) which completely neglects transcytosis ($\lambda_i = 0$) and only involves two Dpp components: diffusing Dpp (external component) and non-diffusing

Dpp (Tkv-bound and internalized components).
Experimental data for GOF experiments (see Fig. 3 in the main paper and supplementary figure S3) show a peak of total Dpp inside the clone. In Section 8 we analyze quantitatively the GOF experiments and infer the relative abundance of the components.

7 A simplified external-bound model

7.1 Introduction

According to the experimental data (see Section 6), transcytosis only affects marginally the total profile and can be neglected. A model involving only diffusing Dpp (external component) and non-diffusion Dpp (Tkv-bound and internalized components) is therefore a good approximation to our problem and simplifies the search for optimal parameters (data quantification).
The equations governing the two components, namely $M_e(x)$ and $M_{bi}(x)$, are obtained from Eqs. (7)-(9) and read

$$-D_e M_e''(x) + \alpha_e M_e(x) = S(x) \tag{21}$$

$$M_{bi}(x) = M_b(x) + M_i(x) = \frac{\alpha_e}{\kappa_{bi}} M_e(x), \tag{22}$$

with $\kappa_{bi} \doteqdot \left(\frac{1}{\kappa} + \frac{1}{\alpha_i}\right)^{-1}$.
We recall that we model *tkv* clones by modifying the number of receptors ($T_0 \to nT_0$ for $x \in \mathcal{C}$) and that both Dpp components are affected. For the external component, the effective degradation α_e (linear loss of external component $\alpha_e M_e(x)$ by binding) is affected and there is an overall increase (respectively decrease) of external Dpp if the number of receptors decreases (respectively increases) inside the clone. The non-diffusing component is proportional to $\alpha_e M_e(x)$, therefore linearly affected by the clone.
The model presented in [3], however, is slightly different since the authors consider that degradation happens externally. As a consequence, at steady state, the clones do not affect the external component but only the non-diffusing one (see Eqs (1) and (2) in [3]).

7.2 Total Dpp profile

Introducing the relative percentage of external component $a \in]0, 1[$, the parameters read $\lambda_e = 0.2L$, $\alpha_e = \frac{s_0}{a}$, $\kappa_{bi} = \frac{s_0}{1-a}$ and the total Dpp profile

affected by clones $\bar{M}_{tot} = \bar{M}_e(x) + \bar{M}_{bi}(x)$ reduces to

$$\begin{aligned}\bar{M}_{tot}(x \notin \mathcal{C}) &= \frac{1}{a}\bar{M}_e(x) \\ \bar{M}_{tot}(x \in \mathcal{C}) &= \left(\frac{n}{a} + (1-n)\right)\bar{M}_e(x)\end{aligned}$$

In Figs. 8 and 9 we present the results for LOF and GOF mutant clones experiments. In **(a)**, there is 10% of external Dpp ($a = 0.1$) and the total Dpp profiles are identical to that in Figs. 5 **(a)** and 5 **(c)**. In **(c)**, there is 80% of external Dpp (cf. Figs. 4 **(a)** and **(c)**) and in **(b)**, we show an intermediate case with $a = 0.45$.
Fig. 9 suggests that the amplitude of the peak at the beginning of the clone depends on the value of a. We therefore show in Fig. 10 the ratio

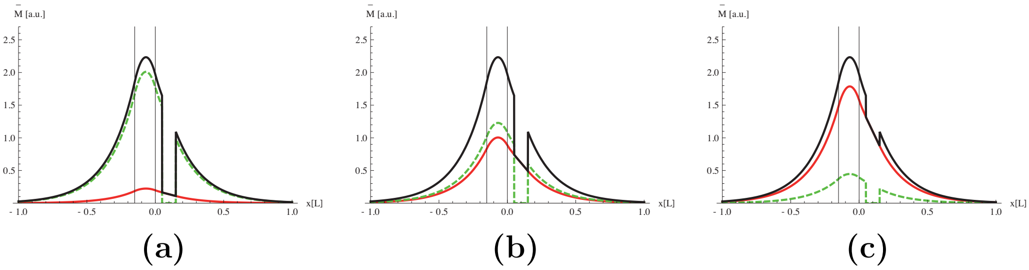

Figure 8: Clone effects on the Dpp profile in the LOF ($n = 0$) case. We show the total Dpp (black solid line) and its external (solid red line) and non-diffusing (dashed green line) components. The source width has been set to $x_0 = 0.15L$ (vertical lines) and the clone is located between $x_1 = 0.05L$ and $x_2 = 0.15L$. The parameters are $\lambda_e = 0.2L$, $\alpha_e = \frac{s_0}{a}$, $\kappa_{bi} = \frac{s_0}{1-a}$. In **(a)** $a = 0.1$, in **(b)** $a = 0.45$ and in **(c)** $a = 0.8$.

$$\rho(n, a) = \frac{\bar{M}_{tot}(x_1^+)}{\bar{M}_{tot}(-(x_0 + x_1))} = c_n\big(n + a(1-n)\big).$$

between the peak inside the clone ($x = x_1^+$) and the level at the opposite position with respect to the source ($x = -x_0 - x_1$) for a ranging from 0.1 to 0.9 and different values of n (from 1 to 19). We note that $c_n = \frac{\bar{M}_e(x_1^+)}{\bar{M}_e(-(x_0+x_1))}$ is $a-$independent, implying that for a given n, $\rho(n, a)$ is linear with a.

In Fig. 11, we show the effect of the clone position for $n = 10$, $a = 0.1$ and x_1 ranging from $0.05L$ to $0.25L$ (clone width of $0.1L$). As expected, $\rho(n, a)$ is strongly affected by the clone position and the ratio increases considerably when the clones are closer to the source.

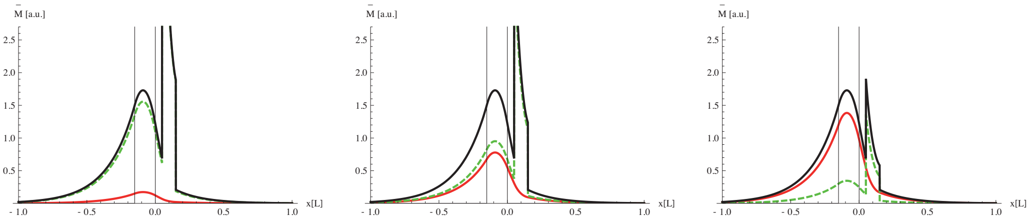

(a) **(b)** **(c)**

Figure 9: Clone effects on the Dpp profile in the GOF ($n = 10$) case. We show the total Dpp (black solid line) and its external (solid red line) and non-diffusing (dashed green line) components. The source width has been set to $x_0 = 0.15L$ (vertical lines) and the clone is located between $x_1 = 0.05L$ and $x_2 = 0.15L$. The parameters are $\lambda_e = 0.2L$, $\alpha_e = \frac{s_0}{a}$, $\kappa_{bi} = \frac{s_0}{1-a}$. In **(a)** $a = 0.1$, in **(b)** $a = 0.45$ and in **(c)** $a = 0.8$.

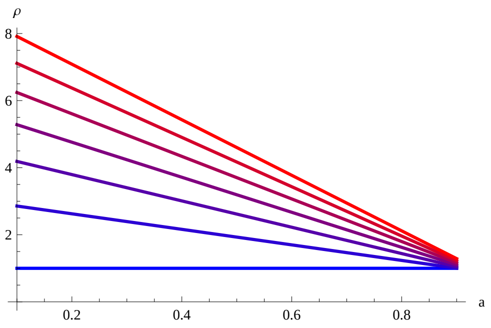

Figure 10: ratio $\rho(n, a)$ between the peak inside the clone and the level at the opposite position with respect to the source for $\bar{M}_{tot}(x)$. n ranges from 1 (blue line, corresponding to the wt) to 19 (red line) and a from 0.1 to 0.9. The source width has been set to $x_0 = 0.15L$ and the clone is located between $x_1 = 0.05L$ and $x_2 = 0.15L$.

8 Quantitative comparison with GOF experiments

Experimental data for GOF experiments (see Fig. 3 in the main paper and supplementary figures S3) show a peak of total Dpp inside the clone. In our model, there are two parameters which are still unknown: n and a. We extracted the total Dpp profile $M(x)$ from each image and calculated the

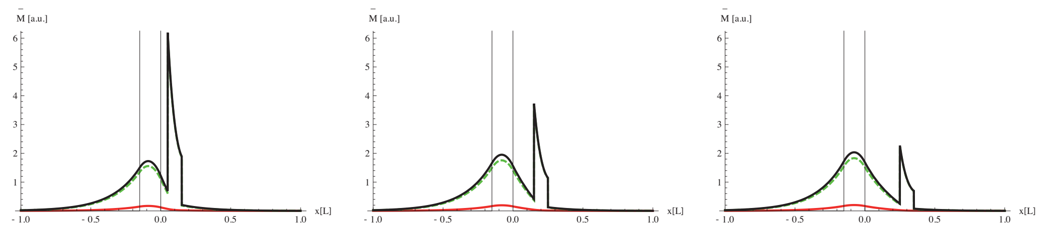

(a) **(b)** **(c)**

Figure 11: Clone effects on the Dpp profile in the GOF ($n = 10$) case. We show the total Dpp (black solid line) and its external (solid red line) and non-diffusing (dashed green line) components. The source width has been set to $x_0 = 0.15L$ (vertical lines). Clone width is $0.1L$ and clone position is $x_1 = 0.05L$ ((**a**)), $x_1 = 0.15L$ ((**b**)) and $x_1 = 0.25L$ ((**c**)). The other parameters are $\lambda_e = 0.2L$, $\alpha_e = 10s_0$, $\kappa_{bi} = \frac{10s_0}{9}$ ($a = 0.1$).

numerical value $\rho^{\text{exp}} = \frac{M(x_1)}{M(-(x_0+x_1))}$ leading to an analytical relationship

$$\rho^{\text{exp}} = \rho(n,a) \Rightarrow a(n) = \left(nc_n - \rho^{\text{exp}}\right) / \left((n-1)c_n\right).$$

The source width x_0 and clone boundaries x_1 and x_2 have been extracted from the image.

In Fig. 12 we show one example of data quantification. In (**a**), the experimental total Dpp profile is shown by GFP antibody staining, filtered with a low Gaussian filter (solid black line). From the Dpp:GFP fluorescence intensity (green line), we estimate the positions of the production region (the vertical blue lines). The vertical red lines show the clone boundaries. The profile has been rescaled as for the analytical profiles and a constant background level has been removed. The value of the normalization constant and shift has been obtained analytically computing an exponential fit (thin blue lines), with decay length $\lambda^{\text{fit}} = 20\mu m$ [2], in front of the clone and after the production region (according to our model, we expect a pure exponential behavior in these regions, see Eq. (19)). In (**b**) we present the corresponding $a(n)$ profile (data quantification). Since we expect at least a ten-fold increase in receptor levels inside the clone, we conclude, in agreement with our qualitative analysis of LOF images (cf. Section 6), that the dominant component of total Dpp is external unbound (at least 80% in this example).

In Fig. 13 we present the $a(n)$ profiles for several GOF images (see Fig. 3 in the main paper and supplementary figures S3). We obtain, according to

our model, that for a ten-fold increase in receptor number inside the clone, around $60-80\%$ of the total Dpp should be external.

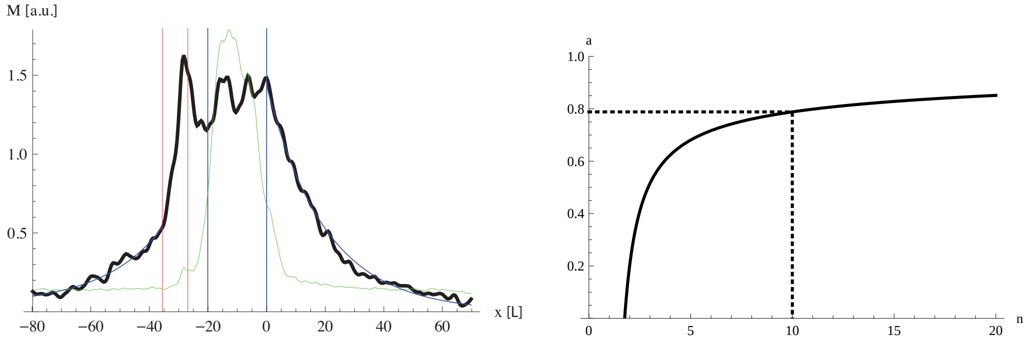

(a) (b)

Figure 12: In (**a**) we present the experimental GFP antibody staining profile, extracted from one image and filtered with a low Gaussian filter (solid black line). The Dpp-GFP fluorescence intensity (green line) shows the positions of the production region (the vertical blue lines). The vertical red lines show the clone boundaries (in this case on the left side of the production region). The thin blue lines in front of the clone and after the production region are obtained by computing an exponential fit and allow to obtain the value of the global background shift. In (**b**) we present the corresponding $a(n)$ profile (data quantification). Since we expect at least a ten-fold increase in receptor levels inside the clone (see vertical dashed line), we expect at least 80% of Dpp to be external and unbound (horizontal dashed line).

9 Study of the non-linear contributions

In this section, we come back to the initial non-linear problem (see Section 2.1) and show that for our particular case (negligible transcytosis, most of the Dpp is external), the linear approximation $T(x) \cong T_0$ is quite accurate.

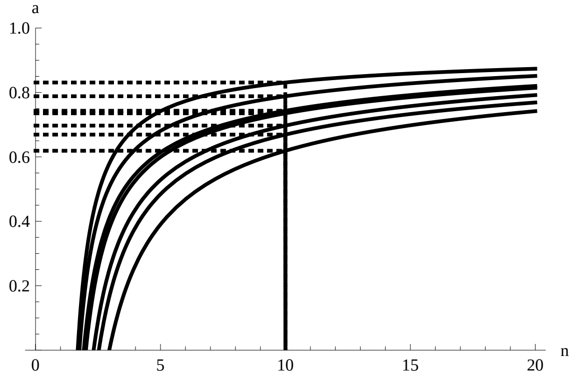

Figure 13: The data quantification $a(n)$ for several GOF images. Dashed lines represent the Dpp external component abundance for a ten-fold increase in receptor concentration inside the clone.

9.1 Numerical resolution

Introducing again the effective diffusion α_e and assuming $D_i = 0$ and $T(x) > 0 \Leftrightarrow T_0 > \max(M_b(x))$, Eqs. (4)-(6) reduce to

$$-\lambda_e^2 \alpha_e M_e''(x) + \frac{\alpha_e M_e(x)}{\frac{\alpha_e}{\kappa_{bi}} \frac{M_e(x)}{\tau_0} + 1} = S(x) \tag{23}$$

$$M_{bi}(x) = \frac{\frac{\alpha_e}{\kappa_{bi}} M_e(x)}{\frac{\alpha_e}{\kappa_{bi}} \frac{M_e(x)}{\tau_0} + 1}. \tag{24}$$

We note that $\tau_0 \doteqdot \frac{\kappa}{\kappa_{bi}} T_0$ appears as a free parameter in the non-linear problem and our linear approximation correspond to the limit $\tau_0 \to \infty$.
For a step source $S(x) = s_0 \theta(x + x_0)\theta(-x)$, the parameters for the linear model (see Section 7) were $\lambda_e = 0.2L$, $\kappa_{bi} = \frac{s_0}{1-a}$, $\alpha_e = \frac{s_0}{a}$, with $a \in]0, 1[$ being the abundance of the external component. In the non-linear model, as the $M_{bi}(x)$ component is not diffusing, the relationship $\kappa = \frac{s_0}{1-a}$ is still valid. Assuming again a constant decay length $\lambda_e = 0.2L$, the remaining two parameters are constrained by the normalization of the external component $I_e(\alpha_e, \tau_0) = a$. The numerical $\alpha_e(\tau_0)$ relationship for the particular case of 70% of external Dpp (i.e. $a = 0.7$) is presented in Fig. 14. However, if we impose negligible levels at the pouch boundaries ($r_{bi} = M_{bi}(-L)/M_{bi}(-x_0/2) <$ 2%, see Fig. 15 **(a)**), we find a minimal limiting value for τ_0: $\tau_0^{min} \cong 2$. We note that τ_0 is expressed in the same arbitrary units as the Dpp profiles. The justification of such a limiting value can be given considering Fig. 15 **(b)**, where we present the maximal amplitude $M_{bi}(-x_0/2)$ as a function of τ_0.

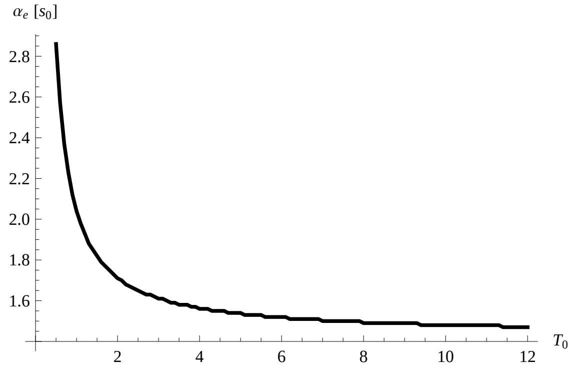

Figure 14: Numerical relationship $\alpha_e(\tau_0)$ ensuring a normalization $I_e(\alpha_e, \tau_0) = a = 0.7$ of the external Dpp component. τ_0 ranges from 0.5 to 12 and the other parameters have been set to $\lambda_e = 0.2L$ and $\kappa_{bi} = \frac{s_0}{1-a} = \frac{s_0}{0.3}$.

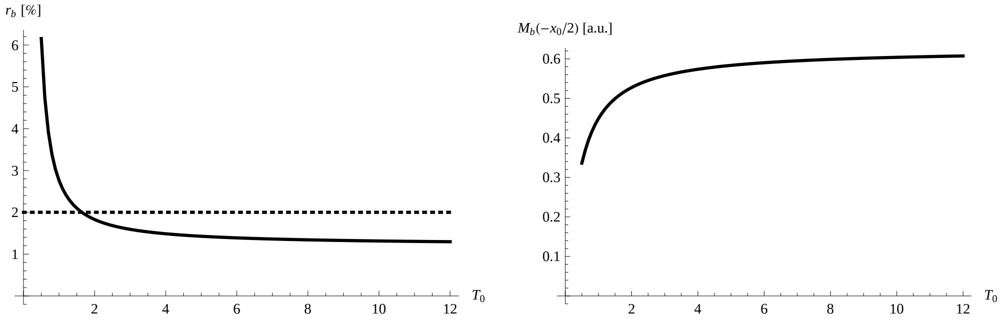

(a) **(b)**

Figure 15: In **(a)** we present the numerical relationship $r_{bi} = M_{bi}(-L)/M_{bi}(-x_0/2)$ and in **(b)** the numerical value of the maximal amplitude $M_{bi}(-x_0/2)$ as function of τ_0 (ranging from 0.5 to 12). The other parameters have been set to $\lambda_e = 0.2L$, $\kappa = \frac{s_0}{1-a} = \frac{s_0}{0.3}$ and $\alpha_e(\tau_0)$ according to Fig. 14. The dotted horizontal line in **(a)** shows the position $r_{bi} = 2\%$ related to a minimal limit value $\tau_0^{min} \cong 2$.

The amplitude of the bound component decreases when τ_0 decreases. As the normalization of the profile is fixed ($I_{bi} = 1 - a$), we therefore expect a flattening of the profile and, for $\tau_0 < 2$, non-negligible levels at the boundaries are reached.

As expected, in Fig. 14, the profile $\alpha_e(\tau_0)$ reaches the asymptotic value $\alpha_e(\infty) = \frac{s_0}{a}$ corresponding to the linear approximation. In Fig. 16, we there-

fore compare the wt, LOF and GOF profiles for the limiting value ($\tau_0 = 2$, dotted lines) and for the asymptotic approximation ($\tau_0 = 100$, solid lines). We clearly see that the profiles are qualitatively very similar, the main difference being inside the source and not related to the clone position, giving *a posteriori* justification to our linear approximation of the problem.
However, we would like to point out that such a numerical study of the non-linear problem starting from an arbitrary set of parameters obtained from the linear model, e.g. for the scenarios where the total Dpp is mainly internalized or mainly bound, is more complicated because additional parameters, namely λ_e and/or λ_i are no longer anymore constrained theoretically. Thus it is harder to obtain a posteriori justification for the other scenarios.

9.2 Power expansion solution

We note that $N-$order better approximated analytical solutions $M_e(x) \cong \sum_{n\geq 0}^{N} \frac{1}{\tau_0^n} M_e^n(x)$ of the differential equation (23) can be found iteratively introducing the power expansion

$$\frac{\alpha_e M_e(x)}{\frac{\alpha_e}{\kappa_{bi}} \frac{M_e(x)}{\tau_0} + 1} = \alpha_e M_e(x) \sum_{n\geq 0} \left(\frac{\alpha_e M_e(x)}{\kappa_{bi} \tau_0} \right)^n .$$

The lower expansion order solution $M_e^0(x)$ is, as expected, the solution (11) of the linear problem. The first order solution reads

$$M_e^1(x) = \frac{\alpha_e^2}{\kappa_{bi}} \int_{\mathbb{R}} dy \, G_e(x-y) \left[M_e^0(y) \right]^2 .$$

In Fig. 17 we compare the analytical solutions $M_e^0(x)$ and $M_e^0(x) + \frac{1}{\tau_0} M_e^1(x)$ to the numerical solution of the non-linear problem for $\tau_0 = 2$. We note that the first order solution is already very accurate. Therefore, higher order analytical solutions allow to optimize the search of optimal parameter values: for a fixed value of τ_0 and a, an analytical relationship $\alpha_e(\tau_0, a, \lambda_e)$ is obtained from the integral I_e. As $\kappa_{bi} = s_0/(1-a)$, the unique remaining free parameter, namely λ_e, can be found imposing an exponential fitting procedure with decay length $\lambda^{\text{fit}} = 0.2L$ for $x > 10\%L$. Assuming $\tau_0 = 2$ and $a = 0.7$, we obtain $\lambda_e \cong 0.192$, $\kappa_{bi} = \frac{10 s_0}{3}$ and $\alpha_e \cong 1.697$.
To conclude, we point out that our approach is not anymore valid for very small values of τ_0 because (i) we always assume $T(x) > 0$ and (ii) the asymptotic expansion may not be valid for small τ_0 values.

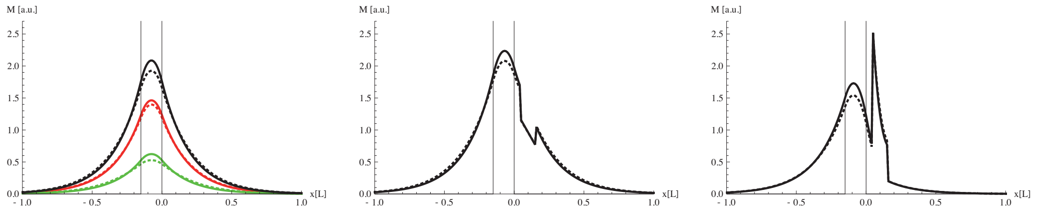

(a) (b) (c)

Figure 16: Comparison between the limit case $\tau_0 = 2$ (dotted lines) and asymptotic case $\tau_0 = 100$ (solid lines) for the wt, LOF and GOF profiles (**(a)**, **(b)** and **(c)** respectively). The black lines represent the total Dpp, the red lines the external components and the green lines the non-diffusing components. The source width has been set to $x_0 = 0.15L$ (vertical lines) and clone position is $x_1 = 0.05L$, $x_2 = 0.15L$. The other parameters are $\lambda_e = 0.2L$, $\kappa_{bi} = \frac{10s_0}{3}$ and $\alpha_e(2) \cong 1.71s_0$, $\alpha_e(100) \cong \frac{10s_0}{7}$.

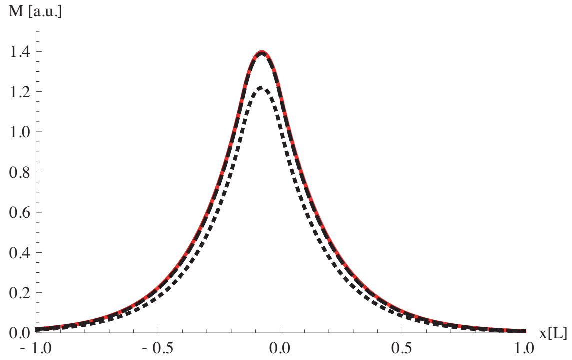

Figure 17: Comparison of the zero and first order solutions $M_e^0(x)$ (black dotted line) and $M_e^0(x) + \frac{1}{\tau_0}M_e^1(x)$ (black dashed line) to the numerical solution of the non-linear problem (red line) for $\tau_0 = 2$, $\lambda_e = 0.2L$, $\kappa_{bi} = \frac{s_0}{1-a} = \frac{s_0}{0.3}$ and $\alpha_e(2) \cong 1.71s_0$.

10 Conclusion

In this supplementary information, we first proposed a model describing the steady state of the total Dpp profile in wt tissues (1D linearized model). We identified three components for the total Dpp: external, bound to the Tkv receptors and internalized. The external Dpp diffuses from a finite-size production region and can bind to the Tkv receptors. The bound Dpp can unbind or be internalized. The internalized Dpp can be degraded or

transported by transcytosis. Within the approximation of a large number of receptors, we obtained an analytical solution for each component (cf. Section 2).

In *tkv* clone experiments, the number of free receptors inside the clonal region is affected leading to an increase or decrease of the effective binding rate. Transcytosis is also affected by the clones because we assume that cell by cell transport is receptor-mediated. In Section 4 we presented explicit analytical expressions for the three Dpp components in the presence of clones.

Our model involves six free parameters. Since it is very hard to determine experimentally the value of these parameters, we studied three limit scenarios corresponding to a total Dpp profile that is mostly external, Tkv-bound or internalized (cf. Section 3). Comparing qualitatively LOF experimental data to our three limit scenarios (cf. Sections 5 and 6), we conclude that transcytosis cannot play a major role in the Dpp gradient formation and that most of the Dpp should be external. Therefore, we proposed a simplified model (cf. Section 7) which only involves diffusing (external) and non-diffusing (Tkv-bound and internalized) Dpp components. GOF experimental data show a peak of Dpp inside the clonal regions. Data extraction (cf. Section 8) based on the quantification of the peak amplitude confirms, according to our model, that most of the Dpp should be external and unbound ($60-80\%$ for a ten-fold increase in receptor levels). In Section 9, we finally give a *a posteriori* justification of our assumption of a large number of free receptors (linearization of the problem) by studying the initial non-linear problem with the explicit set of parameters figured out in the previous sections.

References

[1] T. Bollenbach, K. Kruse, P. Pantazis, M. González-Gaitán, and F. Jülicher, "Morphogen transport in epithelia," *Phys Rev E Stat Nonlin Soft Matter Phys*, vol. 75, p. 011901, Jan 2007.

[2] A. Kicheva, P. Pantazis, T. Bollenbach, Y. Kalaidzidis, T. Bittig, F. Jülicher, and M. González-Gaitán, "Kinetics of morphogen gradient formation," *Science*, vol. 315, pp. 521–525, Jan 2007.

[3] A. Eldar and N. Barkai, "Interpreting clone-mediated perturbations of morphogen profiles," *Dev Biol*, vol. 278, pp. 203–207, Feb 2005.
